# A Hypoxia and Immune Escape-Related Gene Signature for the Diagnosis of Prostate Cancer: An Integrated Bioinformatics Study

**DOI:** 10.7150/ijms.133397

**Published:** 2026-05-18

**Authors:** Hexia Gan, Jiana Jiang, Jiebin Yang, Feng Shen, Weihong Lu, Peng Lai, Lingyu Chen, Zhibing Xu, Jianping Zhang, Jianming Guo, Fan Chao

**Affiliations:** 1 Department of Oncology, Zhongshan Hospital (Xiamen), Fudan University, Xiamen, China.; 2 Clinical Research Center for Precision Medicine of Abdominal Tumor of Fujian Province, Zhongshan Hospital (Xiamen), Fudan University, Xiamen, China.; 3 Xiamen Clinical Research Center for Cancer Therapy, Zhongshan Hospital (Xiamen), Fudan University, Xiamen, China.; 4 Department of Oncology, Zhongshan Hospital, Fudan University, Shanghai, China.; 5 Department of Gynecology, Zhongshan Hospital (Xiamen), Fudan University, Xiamen, China.; 6 Department of Urology, Zhongshan Hospital (Xiamen), Fudan University, Xiamen, China.; 7 Department of Urology, Zhongshan Hospital, Fudan University, Shanghai, China.

**Keywords:** gene signature, hypoxia, immune escape, prostate cancer, diagnosis

## Abstract

Prostate cancer remains a major global health concern for men, with hypoxia and immune escape being key drivers of tumor progression; however, no reliable diagnostic signature integrating both processes has been established for clinical use. In this study, we integrated RNA sequencing and microarray data from The Cancer Genome Atlas (TCGA)-PRAD, Genotype-Tissue Expression (GTEx), and Gene Expression Omnibus (GEO) datasets to identify hypoxia and immune escape-related genes (HIERGs). Through differential expression analysis, functional enrichment, and machine learning algorithms including Random Forest, SVM-RFE, and LASSO regression, we constructed a robust six-gene diagnostic signature comprising *RBMS3*, *ALDH2*, *SLC7A11*, *FGFR2*, *GDF15*, and *NCAM1*. The model demonstrated high diagnostic accuracy, with area under the curve (AUC) values exceeding 0.9 across all validation cohorts, as confirmed by calibration curves, decision curve analysis, and receiver operating characteristic curves. Functional analysis revealed significant enrichment in translation-related pathways and smooth muscle contraction, and the RiskScore was significantly associated with altered immune cell infiltration in the tumor microenvironment. Regulatory network analysis further uncovered potential upstream regulators and therapeutic agents targeting the key genes. In conclusion, we developed and validated a hypoxia and immune escape-related gene signature with robust diagnostic performance for prostate cancer. Exploratory grouping by the model-derived RiskScore revealed differences in pathway and immune infiltration patterns, suggesting an association between the signature and intratumoral molecular heterogeneity. These exploratory findings primarily serve to characterize the biological features related to the model and provide supplementary clues for understanding molecular alterations in prostate cancer; they should be interpreted with caution.

## 1. Introduction

Prostate cancer remains one of the most prevalent malignancies and a leading cause of cancer-related mortality among men worldwide. In high-human development index countries like the United States, about one in seven men is diagnosed with prostate cancer during their lifetime, underscoring its prevalence as the second most common male malignancy 1. Moreover, emerging data from China indicate a rapid rise in prostate cancer incidence and burden, reflecting shifts in cancer profiles associated with aging populations and lifestyle changes 2. While clinical assessments such as prostate-specific antigen (PSA) testing and Gleason scoring are routinely used for diagnosis and molecular stratification, their limitations in specificity and sensitivity often lead to over-diagnosis, under-treatment, or unnecessary invasive procedures. Consequently, there is an urgent need to identify more reliable molecular biomarkers and develop robust diagnostic models to improve early detection and personalized risk assessment.

The tumor microenvironment (TME) plays a critical role in cancer progression and treatment response 3. Within the tumor microenvironment (TME), hypoxia and immune escape represent two pivotal processes that have been extensively implicated in tumorigenesis, metastasis, and therapy resistance 4. Prostate cancer development and progression are closely associated with the tumor microenvironment, in which hypoxia and immune escape are considered key determinants of tumor biological behavior. Hypoxia promotes tumor cell invasiveness and drug resistance by activating the HIF signaling pathway, thereby enhancing angiogenesis, metabolic reprogramming, and epithelial-mesenchymal transition 3. At the same time, it impairs the anti-tumor function of immune cells by inducing the expression of immunosuppressive molecules such as PD-L1 and CD47 4. Immune escape mechanisms further sustain an immunosuppressive microenvironment, leading to persistent tumor growth and reduced responsiveness to immunotherapy. Given the coupling effects of these two processes in prostate cancer, we integrated hypoxia- and immune escape-related genes for systematic analysis, aiming to elucidate their roles in the molecular pathology of prostate cancer and to identify key molecules with diagnostic and potential therapeutic value. Although numerous genes associated with hypoxia and immune escape have been identified, their combined role and clinical utility as an integrated signature in prostate cancer remain poorly explored.

Recent advances in high-throughput sequencing and public genomic databases, such as The Cancer Genome Atlas (TCGA) and the Gene Expression Omnibus (GEO) 5, provide unprecedented opportunities to systematically identify molecular subtypes and biomarker candidates. Moreover, the integration of multi-omics data and the application of machine learning approaches offer promising avenues for constructing diagnostic and prognostic models with improved accuracy and clinical applicability 5.

In this study, we aimed to identify a novel gene signature related to hypoxia and immune escape (HIERGs) and evaluate its diagnostic value in prostate cancer. By leveraging integrated bioinformatic analyses and a multi-step machine learning framework, we developed and rigorously validated a diagnostic model, which identified a concise gene signature with high predictive accuracy for prostate cancer. This model, derived from key genes, demonstrated robust predictive accuracy across multiple independent cohorts. Furthermore, we explored the underlying biological functions, pathway activities, immune infiltration characteristics, and regulatory networks associated with the identified key genes. Our work provides a comprehensive framework for understanding the molecular mechanisms driven by hypoxia and immune escape in prostate cancer and offers a potential tool for enhanced diagnosis and molecular characterization.

## 2. Methods

### 2.1 Data Acquisition

We obtained raw count data from The Cancer Genome Atlas (TCGA) database (https://portal.gdc.cancer.gov/) for the TCGA-Prostate Adenocarcinoma (PRAD) project using the R package TCGAbiolinks 6 (Version 2.30.0). A total of 499 prostate cancer samples and 52 control samples were included as the test cohort. Additionally, 100 normal prostate tissue samples from the Genotype-Tissue Expression (GTEx) database were incorporated as controls. TCGA-PRAD transcriptomic data were processed and converted to a fragments per kilobase per million (FPKM) expression matrix. Both datasets were individually log2-transformed, and then a merged expression matrix was constructed based on shared protein-coding genes annotated with consistent gene symbols for subsequent differential expression analysis, enrichment analysis, and model construction. TCGA-PRAD and GTEx data originate from different public repositories and inherently differ in sample collection, processing workflows, and data generation backgrounds. These datasets were analyzed after harmonizing expression units and gene annotation, and the merged expression matrix was used for subsequent analyses.

The prostate cancer datasets GSE88808 7 and GSE21034^8^ were downloaded from the Gene Expression Omnibus (GEO) database (https://www.ncbi.nlm.nih.gov/geo/) using the R package GEOquery 9. Both datasets were derived from Homo sapiens and prostate tissues. The platform for GSE88808 was GPL22571, and for GSE21034, GPL5188 was selected (details in Table 1). From GSE88808, 49 prostate cancer samples and 49 normal samples were included as prostate cancer and control groups, respectively. From GSE21034, 131 primary prostate cancer samples and 29 normal samples under platform GPL5188 were selected as prostate cancer and control groups. All selected samples were included in subsequent analyses.

To obtain a candidate gene set related to hypoxia and immune escape, we first retrieved relevant genes from the GeneCards database 10 (https://www.genecards.org/) using the keywords “Hypoxia” and “Immune Escape” as an initial candidate collection. Since GeneCards integrates multi-source literature and algorithm-based association scores, we further performed cross-screening and redundancy removal by combining previously published gene sets and those from other public databases to enhance the biological specificity and reliability of the screened results. Through a PubMed search using the same keywords, we obtained published gene sets including 5 hypoxia-related genes (FRGs) 11 and 4 IERGs 12. After merging and removing duplicates, a total of 6,360 HRGs and 761 IERGs were retained (see Table S1).

The GSE88808 and GSE21034 datasets were each normalized, probe-annotated, and log_2_-transformed using the R package limma. The preprocessed GTEx_TCGA-PRAD merged dataset was used as the test set, while the independently processed GSE88808 and GSE21034 datasets served as validation sets 13.

### 2.2 Differential Gene Expression Analysis

Based on the sample groupings in the merged prostate dataset (GTEx_TCGA-PRAD) and datasets GSE88808 and GSE21034, samples were classified into prostate cancer and control groups, respectively. Differential expression analysis between the PRAD and control groups was performed using the R package limma (Version 3.58.1). Genes with |logFC| > 0.5 and a *p*-value < 0.05 were defined as differentially expressed genes (DEGs). Specifically, genes with logFC > 0.5 and *p*-value < 0.05 were considered up-regulated, while those with logFC < -0.5 and *p*-value < 0.05 were deemed down-regulated. The results of the differential expression analysis were visualized using volcano plots generated with the R package ggplot2 (Version 3.4.4).

To identify hypoxia and immune escape-related differentially expressed genes (HIERDEGs) associated with prostate cancer, we intersected all DEGs (|logFC| > 0.5 and *p*-value < 0.05) obtained from the differential analysis of the merged prostate dataset (GTEx_TCGA-PRAD), GSE88808, and GSE21034 with the previously collected HIERGs. Venn diagrams were plotted to illustrate the overlaps. The resulting HIERDEGs were further visualized in heatmaps using the R package pheatmap (Version 1.0.12).

### 2.3 Gene Ontology Analysis

Gene Ontology (GO) analysis 14 is a widely used method for large-scale functional enrichment studies, encompassing three categories: Biological Process (BP), Cellular Component (CC), and Molecular Function (MF). We performed GO analysis on the HIERDEGs using the R package clusterProfiler^15^ (Version 4.10.0). Terms with a *p*-value < 0.05 and a false discovery rate (FDR, *q*-value) < 0.25 were considered statistically significant.

### 2.4 Gene Set Enrichment Analysis

Gene Set Enrichment Analysis (GSEA) 16 is a computational method that determines whether a predefined set of genes shows statistically significant, concordant differences between two biological states. In this study, genes from the merged prostate dataset (GTEx_TCGA-PRAD) and GSE88808 were first ranked based on their logFC values between the prostate cancer and control groups. GSEA was then performed on all genes in the merged prostate dataset (GTEx_TCGA-PRAD) using the R package clusterProfiler^15^ (Version 4.10.0). The following parameters were applied: seed = 2020, minimum gene set size (minSize) = 10, and maximum gene set size (maxSize) = 500. The gene set c2.all.v2023.2.Hs.symbols was obtained from the Molecular Signatures Database (MSigDB) for the analysis. Significantly enriched gene sets were selected using an adjusted *p*-value (adj.*p*) < 0.05 and a FDR (*q*-value) < 0.25, with *p*-value adjustment performed via the Benjamini-Hochberg (BH) method.

### 2.5 Gene Set Variation Analysis

Gene Set Variation Analysis (GSVA)^17^ is a non-parametric and unsupervised method that transforms a gene expression matrix into a gene set enrichment matrix across samples, enabling the assessment of pathway activity and functional enrichment in transcriptomic data. In this study, the gene set h.all.v7.4.symbols.gmt was obtained from the MSigDB^18^. Using the R package GSVA (Version 1.50.0), we performed GSVA on all genes from prostate cancer samples in the merged dataset (GTEx_TCGA-PRAD) and the GSE88808 dataset. This analysis quantified enrichment scores for each gene set between the prostate cancer and control groups. Gene sets with a *p*-value < 0.05 were considered statistically significant.

### 2.6 Construction of a Prostate Cancer Diagnostic Model

To construct a diagnostic model for prostate cancer, the 15 HIERDEGs identified from the merged dataset (GTEx_TCGA-PRAD) were subjected to a multi-step feature selection pipeline. First, the Random Forest (RF) algorithm was applied to rank the genes by importance, and the top 10 genes were retained. Subsequently, support vector machine recursive feature elimination (SVM-RFE)^19^ algorithm, implemented via the R package e1071 (Version 1.7-14), was applied to the top 10 genes retained from the RF step to identify potential biomarker genes. SVM-RFE is a feature selection method based on support vector machines (SVM) that recursively removes features with the smallest contributions to the classification task, thereby identifying the most informative genes.

Finally, least absolute shrinkage and selection operator (LASSO) regression was performed using the R package glmnet^20^ (Version 4.1-8) on the HIERDEGs selected by the SVM-RFE model. The analysis was conducted with parameters set to set.seed(500) and family = "binomial". LASSO regression introduces an L1 penalty term (lambda × absolute value of coefficients) to conventional linear regression, reducing overfitting and improving the model's generalizability. The results of the LASSO regression were visualized using diagnostic model plots and variable trajectory plots. The final prostate cancer diagnostic model derived from LASSO regression comprised key HIERDEGs referred to as key genes. Based on the regression coefficients from the LASSO model, a RiskScore was calculated for each sample using the following formula:



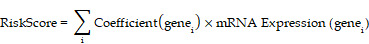



### 2.7 Validation of the Prostate Cancer Diagnostic Model

A nomogram 18 was constructed to visualize the relationships among the key genes based on the results of the LASSO regression analysis, using the R package rms (Version 6.7-1). The nomogram represents multiple independent variables and their functional associations via non-overlapping line segments in a two-dimensional coordinate system. To evaluate the accuracy and discriminative ability of the prostate cancer diagnostic model derived from the LASSO regression, a calibration curve was generated through calibration analysis. Additionally, decision curve analysis (DCA) 21 was performed using the R package ggDCA (Version 1.1) to assess the clinical utility of the key genes within the merged prostate dataset (GTEx_TCGA-PRAD). DCA provides a straightforward method for evaluating clinical prediction models, diagnostic tests, and molecular markers.

Finally, receiver operating characteristic (ROC) curves were plotted and the area under the curve (AUC) values were computed using the R package pROC (Version 1.18.5) for the merged dataset (GTEx_TCGA-PRAD) as well as for the validation sets GSE88808 and GSE21034. These analyses aimed to assess the diagnostic efficacy of the LASSO-derived RiskScore in predicting the occurrence of prostate cancer.

### 2.8 Validation of Expression Differences in Key Genes

To further investigate the expression differences of the Key Genes between the prostate cancer and control groups in the merged prostate dataset (GTEx_TCGA-PRAD) and the validation datasets GSE88808 and GSE21034, ROC curves were generated based on the expression levels of these Key Genes using the R package pROC. The AUC was calculated to evaluate the diagnostic performance of each Key Gene in predicting prostate cancer. The AUC values of ROC curves range from 0.5 to 1. An AUC value closer to 1 indicates better diagnostic performance. Specifically, an AUC between 0.5 and 0.7 suggests low accuracy, between 0.7 and 0.9 indicates moderate accuracy, and above 0.9 represents high accuracy.

### 2.9 Gene Set Enrichment Analysis in High- and Low-Score Groups

Gene Set Enrichment Analysis (GSEA)^16^ was employed to evaluate whether predefined gene sets exhibit statistically significant differences between high- and low-score groups. In this study, genes in the merged prostate dataset (GTEx_TCGA-PRAD) were first ranked based on logFC values between the high- and low-score groups. GSEA was then performed on all genes using the R package clusterProfiler^15^ (Version 4.10.0) with the following parameters: seed = 2020, minimum gene set size (minSize) = 10, and maximum gene set size (maxSize) = 500. The gene set c2.all.v2023.2.Hs.symbols was obtained from the MSigDB for the analysis. Significantly enriched gene sets were identified using an adjusted p-value (adj.*p*) < 0.05 and an FDR (*q*-value) < 0.25, with p-value adjustment performed using the Benjamini-Hochberg (BH) method.

### 2.10 Gene Set Variation Analysis in High- and Low-Score Groups

Gene Set Variation Analysis 17 is a non-parametric, unsupervised method that converts a gene-level expression matrix into a gene set-level enrichment matrix, allowing assessment of pathway activity and functional enrichment in transcriptomic data. The method evaluates whether specific biological pathways are enriched across different sample groups. In this study, the gene set h.all.v7.4.symbols.gmt was obtained from the MSigDB^22^. Using the R package GSVA (Version 1.50.0), we performed GSVA on all genes from prostate cancer samples in the merged dataset (GTEx_TCGA-PRAD) to quantify enrichment scores for each gene set between the high- and low-score groups. Gene sets with a *p*-value < 0.05 were considered statistically significantly enriched.

### 2.11 Immune Infiltration Analysis in High- and Low-Score Groups

Single-sample gene set enrichment analysis (ssGSEA)^17^ was applied to quantify the relative abundance of immune cell infiltration in each sample. First, marker gene sets for various human immune cell subtypes—such as activated CD8+ T cells, activated dendritic cells, gamma-delta T cells, natural killer cells, and regulatory T cells 23—were selected. Using ssGSEA, an enrichment score was computed for each immune cell type in each sample, resulting in an immune infiltration matrix for the merged prostate dataset (GTEx_TCGA-PRAD).

The differences in immune cell infiltration between high- and low-score groups within the prostate cancer cohort were visualized using boxplots generated with the R package ggplot2 (Version 3.4.4). Immune cell types showing significant differences between the two groups were selected for further analysis.

Correlations among the significantly different immune cells were evaluated using Spearman's rank correlation method and visualized in a heatmap via the R package pheatmap (Version 1.0.12). Additionally, correlations between model genes and immune cells were also calculated using Spearman's method and displayed in a bubble plot using ggplot2.

### 2.12 Protein-protein interaction and Regulatory Network Analysis of Key Genes

A protein-protein interaction (PPI) network represents physical and functional interactions between proteins, playing crucial roles in biological processes such as signal transduction, regulation of gene expression, energy and material metabolism, and cell cycle control. Systematically analyzing these interactions helps elucidate protein functions within biological systems, uncover reaction mechanisms under disease conditions, and interpret functional associations among proteins.

The STRING database (https://string-db.org/) was used to retrieve both known and predicted interactions involving the Key Genes, with a minimum required interaction score set to 0.150. The resulting network was visualized using Cytoscape (version 3.9.1).

Gene Ontology (GO) 14 provides a computational framework for measuring functional similarities between genes and gene sets, forming a basis for many bioinformatic methods. Functional similarity among the Key Genes was assessed using the R package GOSemSim 24 (Version 2.28.0) through "Friends" analysis, which evaluates semantic similarities based on GO annotations.

Transcription factors (TFs) regulate gene expression at the transcriptional level by binding to target gene sequences. Using the ChIPBase database 25 (http://rna.sysu.edu.cn/chipbase/), TFs potentially regulating the Key Genes were identified. The regulatory interactions between TFs and Key Genes were visualized as an mRNA-TF regulatory network using Cytoscape 26.

Additionally, miRNAs play crucial regulatory roles in biological development and evolution by targeting multiple genes, while a single target gene might also be modulated by multiple miRNAs. To explore the relationships between Key Genes and miRNAs, miRNA interactions associated with the Key Genes were predicted using the StarBase v3.0 database 27 (https://starbase.sysu.edu.cn/). The resulting regulatory network between mRNAs and miRNAs (mRNA-miRNA regulatory network) was visualized using Cytoscape.

RNA-binding proteins (RBPs) 28 are key regulators involved in numerous post-transcriptional processes, including RNA synthesis, alternative splicing, modification, transport, and translation. Based on the StarBase v3.0 database 27 (https://starbase.sysu.edu.cn/), RBPs targeting the Key Genes were predicted. The interactions between mRNAs and RBPs (mRNA-RBP regulatory network) were also visualized using Cytoscape.

Finally, the Comparative Toxicogenomics Database (CTD) 29 (https://ctdbase.org/) was utilized to predict both direct and indirect drug targets related to the Key Genes, enabling the investigation of interactions between Key Genes and therapeutic compounds. An mRNA-drug regulatory network was constructed and visualized using Cytoscape to illustrate these relationships.

### 2.13 Statistical Analysis

All data processing and statistical analyses in this study were performed using R software (Version 4.2.2). Unless otherwise stated, comparisons between two groups of continuous variables were conducted using the Student's *t*-test for normally distributed data and the Mann-Whitney *U* test (Wilcoxon rank-sum test) for non-normally distributed variables. For comparisons among three or more groups, the Kruskal-Wallis test was applied. Correlations between different molecules were assessed using Spearman's correlation analysis. All reported *p*-values are two-sided, and a *p*-value < 0.05 was considered statistically significant.

## 3. Results

### 3.1 Technical Flowchart

**Figure 1:**
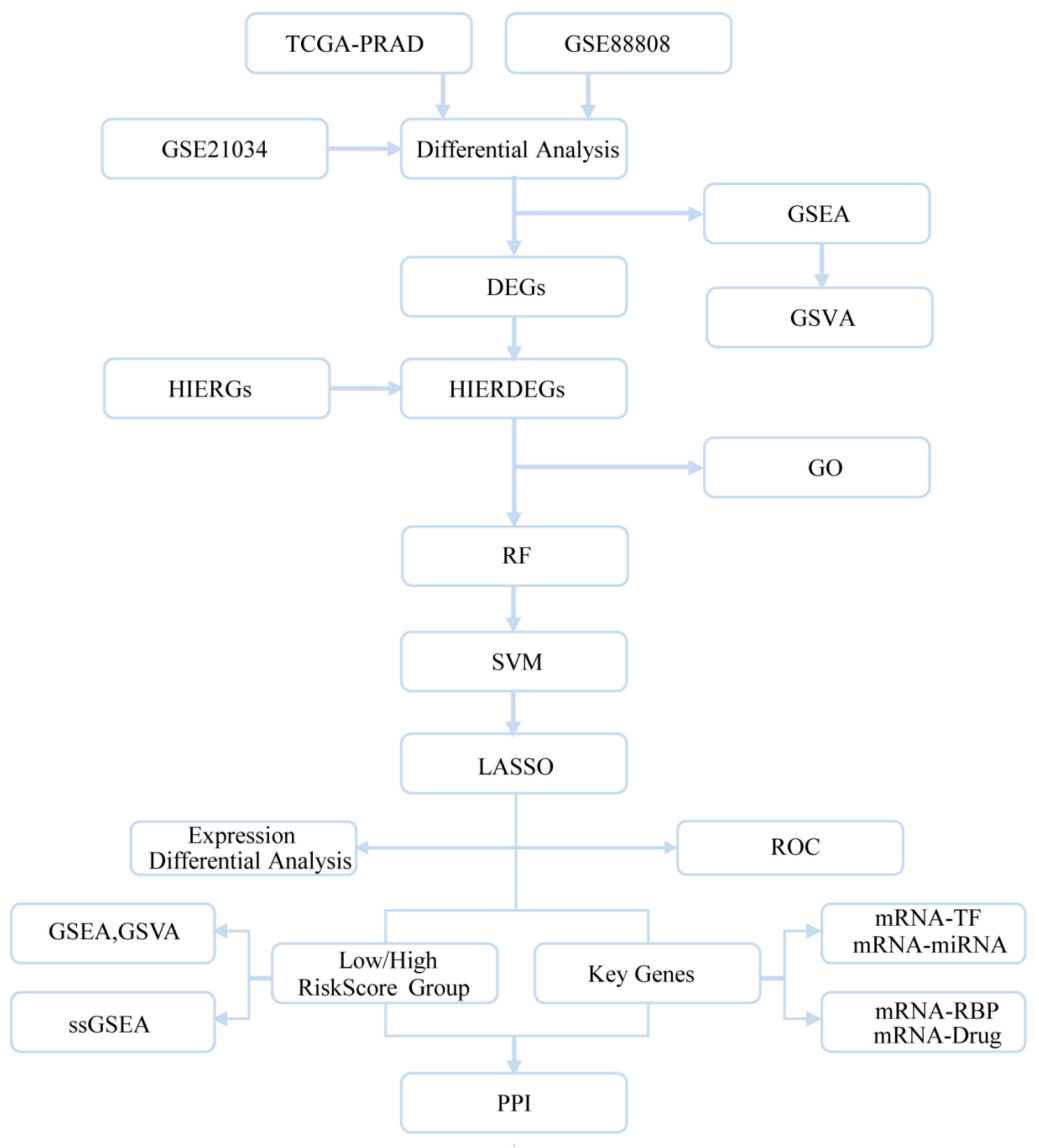
** Technical flowchart.** DEGs, Differentially Expressed Genes; HIERGs, Hypoxia and Immune Escape- Related Genes; HIERDEGs, Hypoxia and Immune Escape-Related Differentially Expressed Genes; GSEA, Gene Set Enrichment Analysis; GO, Gene Ontology; RF, Random Forest; SVM, Support Vector Machine; LASSO, Least Absolute Shrinkage and Selection Operator; TFs, Transcription Factors; RBP, RNA-Binding Protein; GSVA, Gene Set Variation Analysis.

The overall design and workflow of this study are summarized in the technical flowchart (Fig. 1).

### 3.2 Data Standardization of Prostate Cancer Datasets

**Figure 2:**
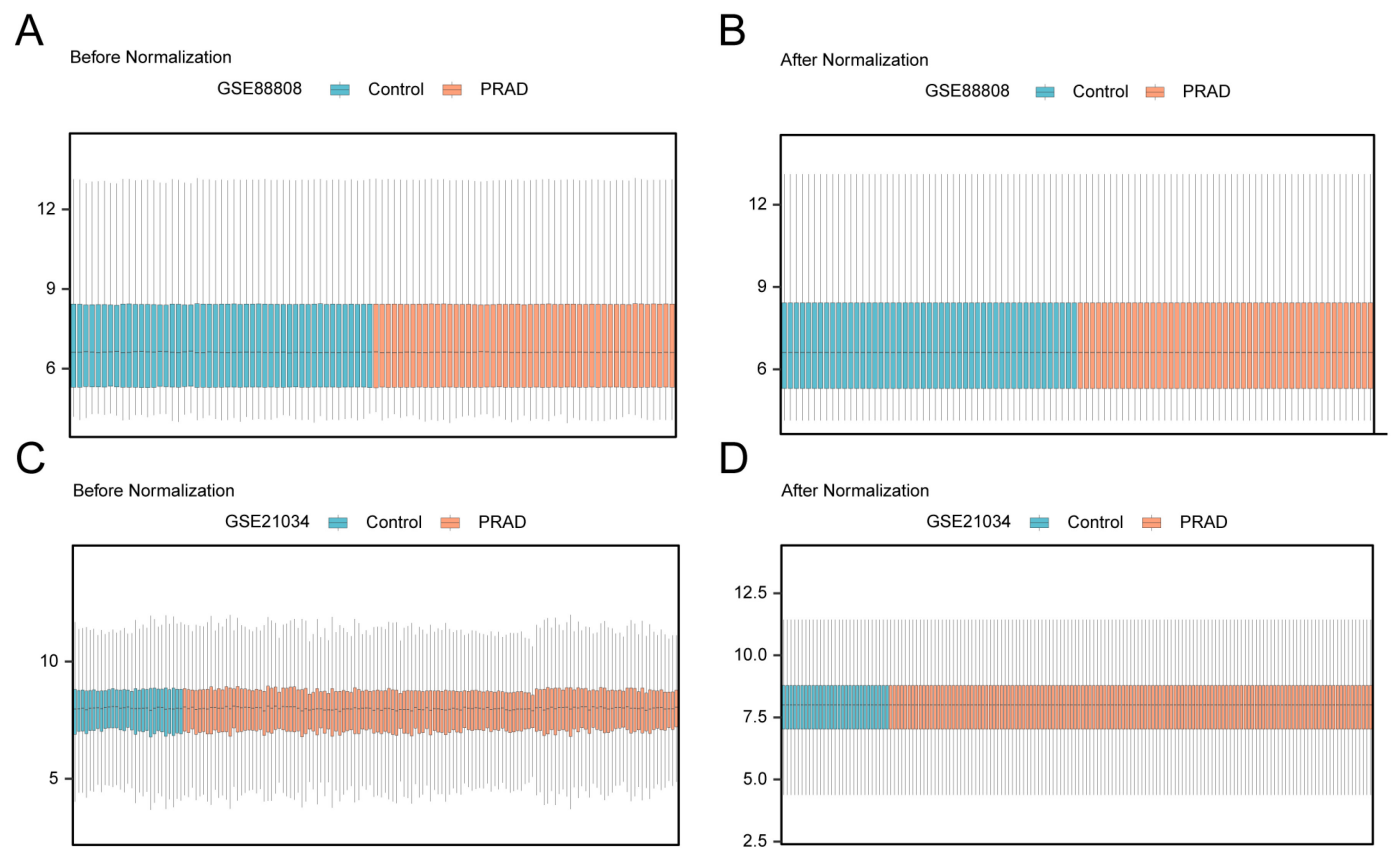
** Data standardization.** (A) Distribution boxplot of dataset GSE88808 before standardization. (B) Distribution boxplot of dataset GSE88808 after standardization. (C) Distribution boxplot of dataset GSE21034 before standardization. (D) Distribution boxplot of dataset GSE21034 after standardization. PRAD, Prostate adenocarcinoma. Orange boxes represent prostate adenocarcinoma (PRAD) samples; blue boxes represent control samples.

Prior to downstream analyses, each dataset underwent expression matrix organization and standardization. Distribution boxplots were used to visualize the expression profiles before and after standardization. Fig. 2A and 2B display the expression distributions of GSE88808 before and after standardization, while Fig. 2C and 2D show the corresponding comparisons for GSE21034. After standardization, the overall expression distributions were reasonably aligned across samples, and the datasets were then used for differential expression and subsequent functional analyses.

### 3.3 Hypoxia and Immune Escape-Related Differentially Expressed Genes in Prostate Cancer

**Figure 3:**
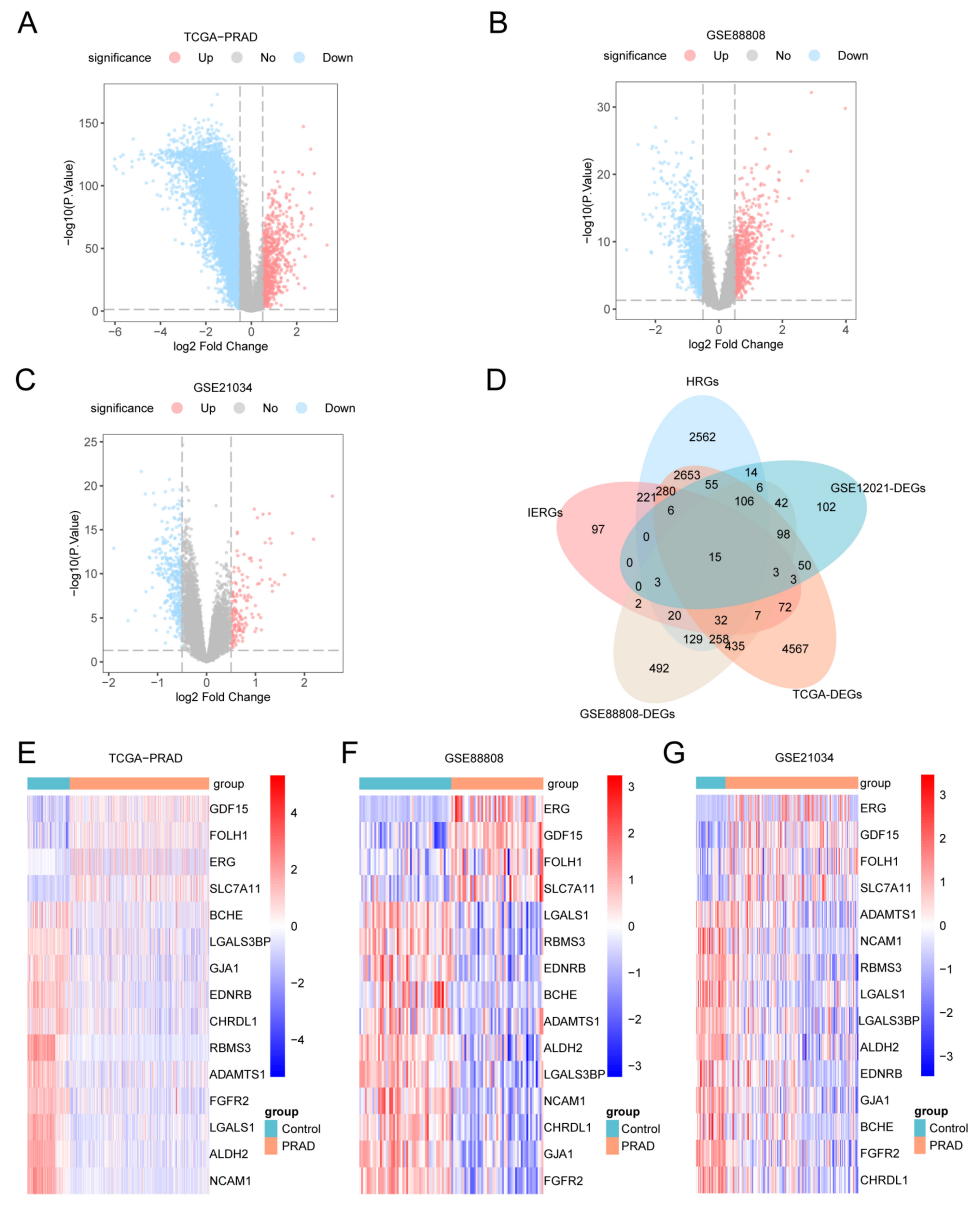
** Differential gene expression analysis.** (A-C) Volcano plots of differentially expressed genes (DEGs) between prostate adenocarcinoma (PRAD) and control groups in the merged GTEx_TCGA-PRAD dataset (A), GSE88808 (B), and GSE21034 (C). (D) Venn diagram showing the intersection between DEGs from all prostate cancer samples and hypoxia and immune escape-related genes (HIERGs) across the merged GTEx_TCGA-PRAD, GSE21034, and GSE88808 datasets. (E-G) Heatmaps of hypoxia and immune escape-related differentially expressed genes (HIERDEGs) in the merged GTEx_TCGA-PRAD (E), GSE88808 (F), and GSE21034 (G) datasets. DEGs, Differentially Expressed Genes; HIERGs, Hypoxia and Immune Escape-Related Genes; HIERDEGs, Hypoxia and Immune Escape-Related Differentially Expressed Genes. Orange indicates the PRAD group; blue indicates the control group. In the heatmaps, red represents high expression and blue represents low expression.

Differential expression analysis between prostate cancer and control groups was performed on the merged GTEx_TCGA-PRAD dataset, GSE88808, and GSE21034 using the R package limma. In the merged GTEx_TCGA-PRAD dataset, a total of 8,640 DEGs met the threshold criteria of |logFC| > 0.5 and p-value < 0.05, comprising 744 up-regulated and 7,896 down-regulated genes, with results visualized in a volcano plot (Fig. 3A). Similarly, the GSE88808 dataset yielded 1,648 DEGs under the same threshold, including 655 up-regulated and 993 down-regulated genes, as shown in Fig. 3B. In the GSE21034 dataset, 503 DEGs were identified, of which 165 were up-regulated and 338 were down-regulated, with the corresponding volcano plot displayed in Fig. 3C.

To identify the HIERDEGs, we intersected all DEGs meeting the threshold (|logFC| > 0.5 and *p*-value < 0.05) from the prostate cancer samples with the previously defined HIERGs. A Venn diagram illustrating the overlap is shown in Fig. 3D. This analysis revealed 15 HIERDEGs (see Table S2 for details). The expression patterns of these 15 HIERDEGs across different sample groups in the merged GTEx_TCGA-PRAD, GSE88808, and GSE21034 datasets were further visualized using heatmaps generated with the R package pheatmap, as shown in Fig. 3E, 3F, and 3G, respectively.

### 3.4 Gene Ontology Analysis

To further investigate the biological roles of the 15 HIERDEGs in prostate cancer, Gene Ontology (GO) enrichment analysis was performed, focusing on biological processes (BP), cellular components (CC), and molecular functions (MF). The complete results are provided in Table 2. The analysis revealed that the 15 HIERDEGs were significantly enriched in several GO terms. Key biological processes included oligopeptide transmembrane transport, positive regulation of smooth muscle cell proliferation, oligopeptide transport, ammonium ion metabolic process, and modified amino acid transport. Enriched cellular components comprised the collagen-containing extracellular matrix, endoplasmic reticulum lumen, blood microparticle, nuclear envelope lumen, and fascia adherens. Molecular functions included modified amino acid transmembrane transporter activity, sulfur compound transmembrane transporter activity, peptide binding, amide binding, and carboxylic ester hydrolase activity.

**Figure 4:**
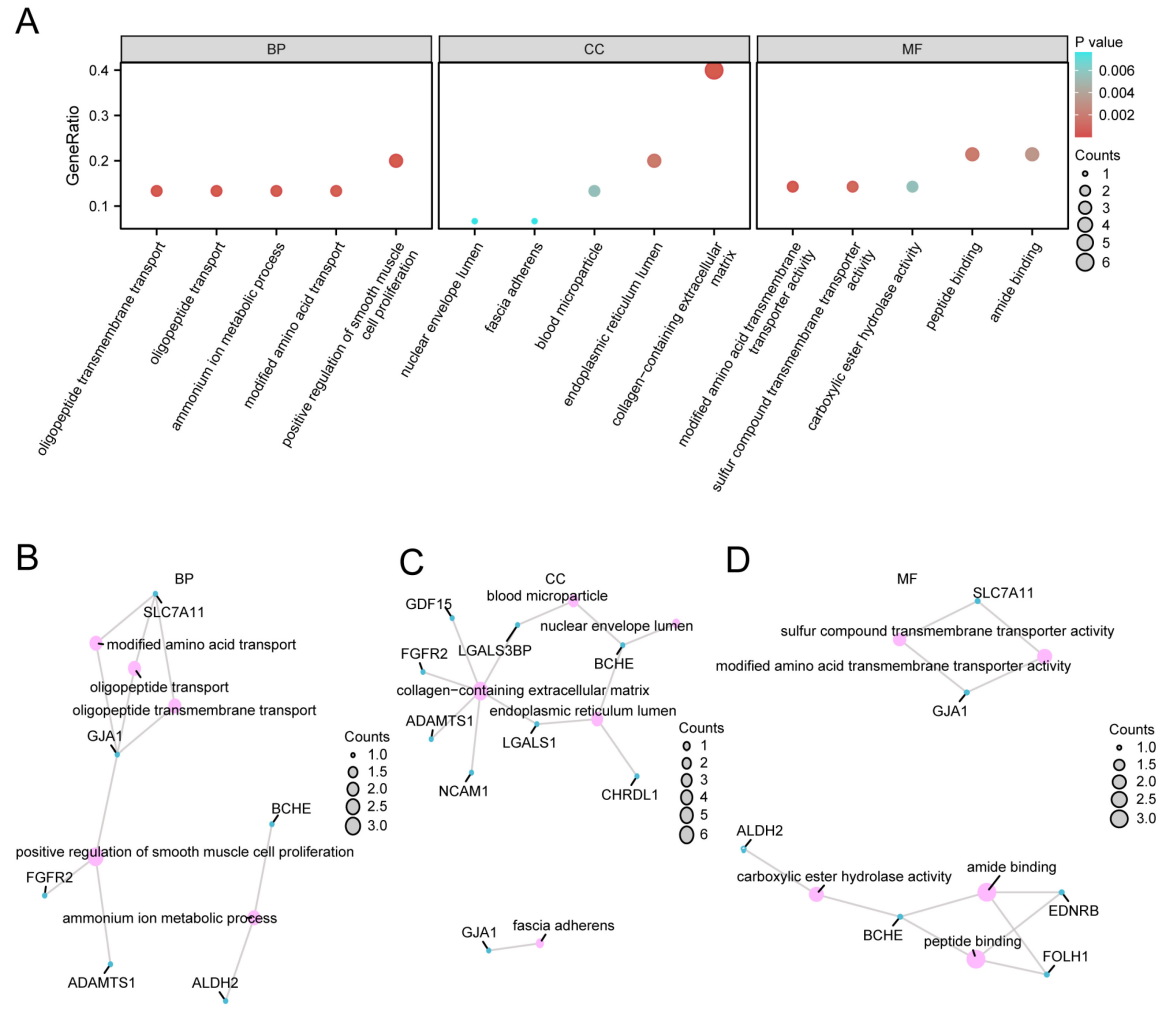
** GO enrichment analysis of HIERDEGs.** (A) Bar plot of Gene Ontology (GO) enrichment analysis for hypoxia and immune escape-related differentially expressed genes (HIERDEGs), showing significantly enriched terms in Biological Process (BP), Cellular Component (CC), and Molecular Function (MF). The x-axis represents the GO terms. (B-D) Network diagrams of the GO analysis results for HIERDEGs: BP (B), CC (C), and MF (D). Light pink nodes represent GO terms; light blue nodes represent molecules; edges represent the association between terms and molecules. HIERDEGs, Hypoxia and Immune Escape-Related Differentially Expressed Genes; GO, Gene Ontology; BP, Biological Process; CC, Cellular Component; MF, Molecular Function. The GO analysis used a significance threshold of *p*-value < 0.05 and false discovery rate (FDR, *q*-value) < 0.25.

The GO enrichment results are summarized in a bar plot (Fig. 4A). Additionally, network diagrams were constructed to visualize the relationships between the HIERDEGs and the significantly enriched terms in BP, CC, and MF (Fig. 4B-D). In these networks, edges represent associations between genes and GO terms, and the node size corresponds to the number of genes annotated to each term.

### 3.5 Gene Set Enrichment Analysis

**Figure 5:**
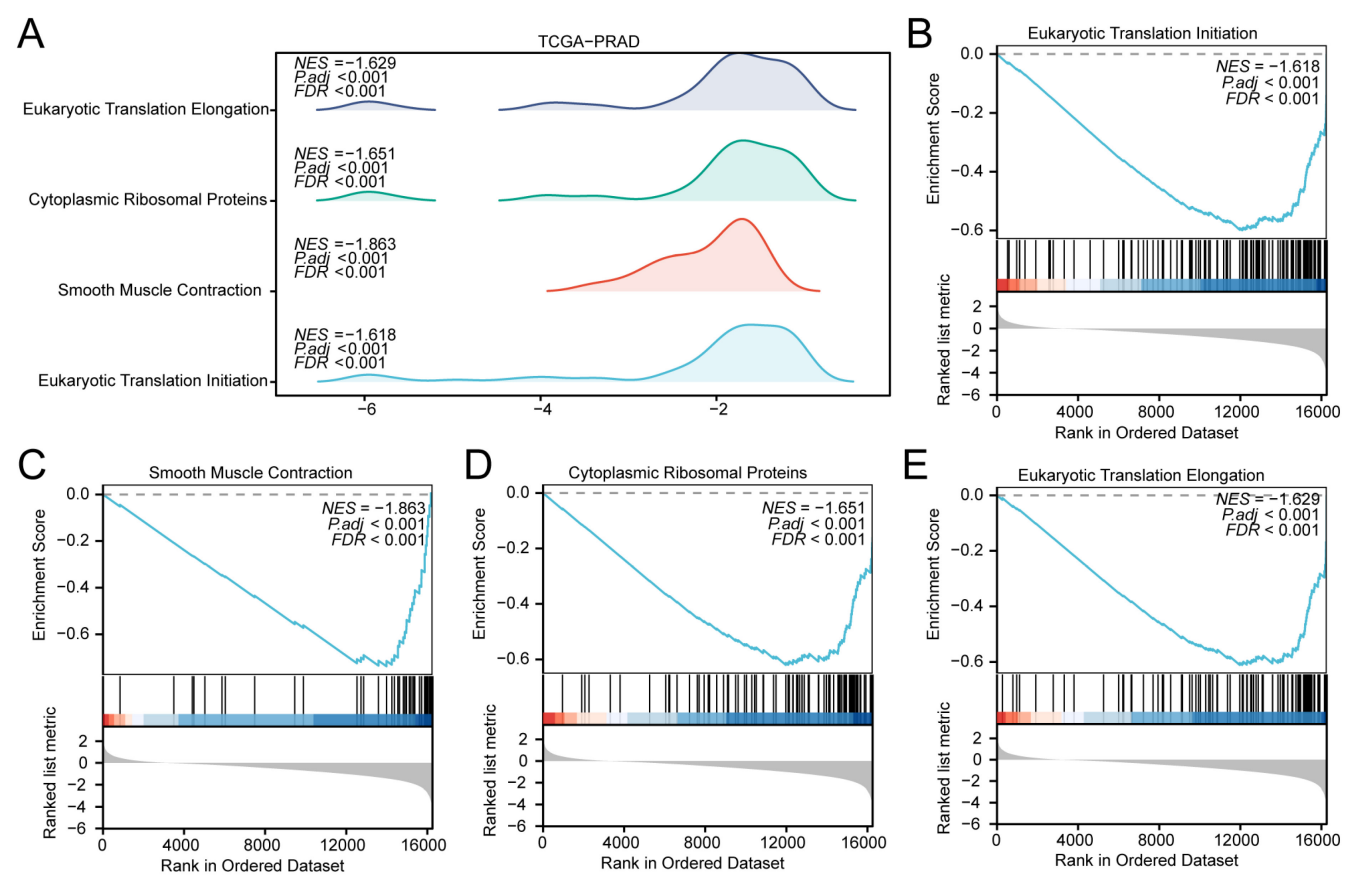
** GSEA of the TCGA-PRAD dataset.** (A) Mountain plot from the Gene Set Enrichment Analysis (GSEA) showing four significantly enriched biological functions in the merged prostate dataset (GTEx_TCGA-PRAD). (B-E) GSEA plots demonstrating significant enrichment of all genes in Eukaryotic Translation Initiation (B), Smooth Muscle Contraction (C), Cytoplasmic Ribosomal Proteins (D), and Eukaryotic Translation Elongation (E). GSEA, Gene Set Enrichment Analysis. Significantly enriched gene sets were selected using an adjusted *p*-value (adj.*p*) < 0.05 and a false discovery rate (FDR, *q*-value) < 0.25, with *p*-value adjustment performed using the Benjamini-Hochberg (BH) method.

To investigate the impact of global gene expression profiles on prostate cancer pathogenesis in the merged GTEx_TCGA-PRAD dataset, GSEA was performed based on the logFC values of all genes between the prostate cancer and control groups. This analysis aimed to identify associations between gene expression and involvement in biological processes, cellular components, and molecular functions. The results are summarized in a mountain plot (Fig. 5A) and detailed in Table 3.

The GSEA identified several pathways with significant enrichment differences between the PRAD and control groups. In the TCGA-PRAD dataset, Eukaryotic Translation Initiation (Fig. 5B; *NES* = -1.618), Smooth Muscle Contraction (Fig. 5C; *NES* = -1.863), Cytoplasmic Ribosomal Proteins (Fig. 5D; *NES* = -1.651), and Eukaryotic Translation Elongation (Fig. 5E; *NES* = -1.629) were enriched in the control group.

**Figure 6:**
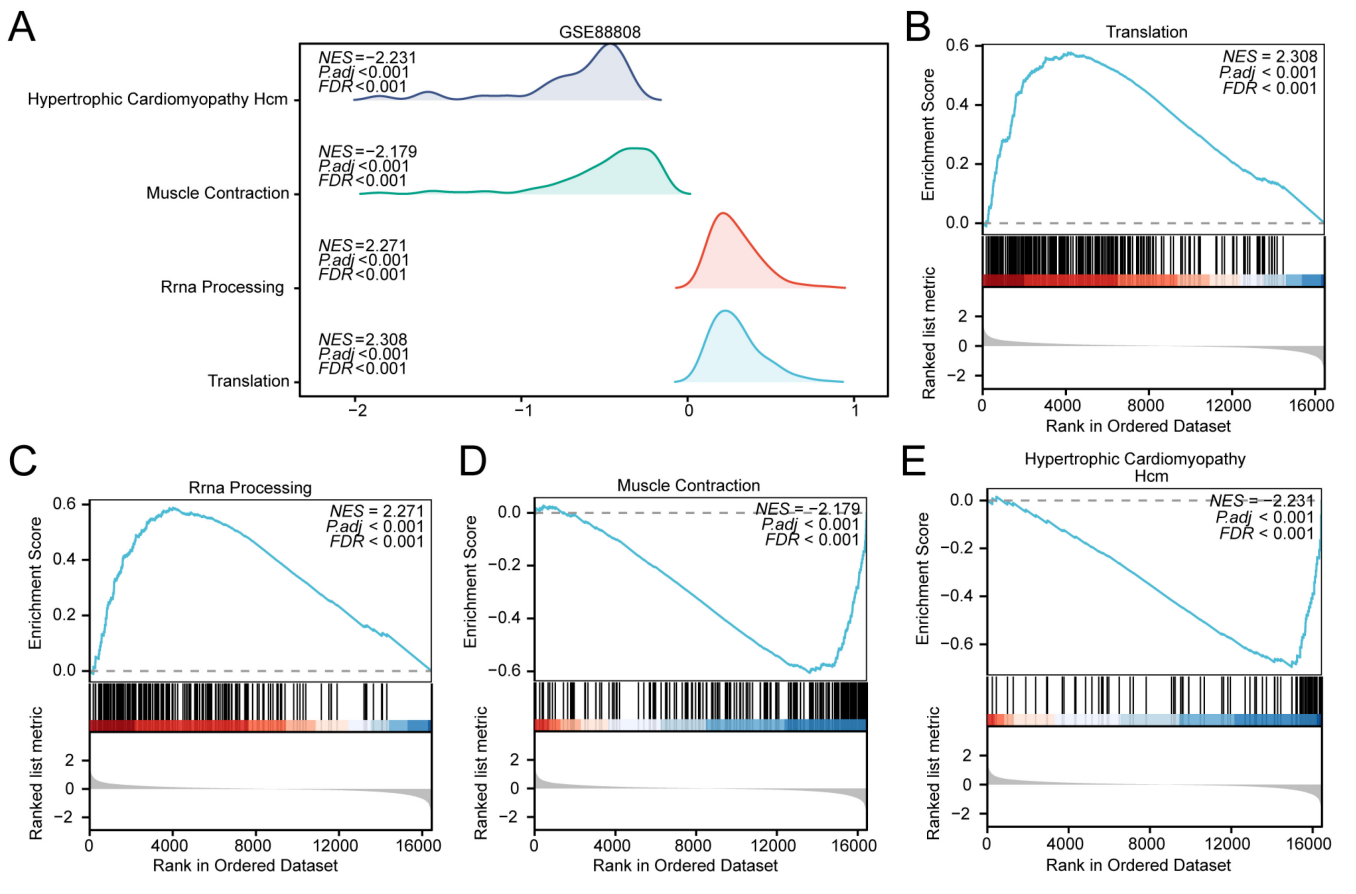
** GSEA of the GSE88808 dataset.** (A) Mountain plot from the Gene Set Enrichment Analysis (GSEA) showing four significantly enriched biological functions in the GSE88808 dataset. (B-E) GSEA plots demonstrating significant enrichment of all genes in Translation (B), rRNA Processing (C), Muscle Contractions (D), and Hypertrophic Cardiomyopathy Hcm (E). GSEA, Gene Set Enrichment Analysis. Significantly enriched gene sets were selected using an adjusted p-value (adj.p) < 0.05 and a false discovery rate (FDR, *q*-value) < 0.25, with p-value adjustment performed using the Benjamini-Hochberg (BH) method.

Similarly, to evaluate the influence of genome-wide expression on prostate cancer development in the GSE88808 dataset, GSEA was conducted using logFC values derived from comparisons between prostate cancer and control samples. The associations between gene expression and functional annotations are presented in a mountain plot (Fig. 6A) and listed in detail in Table 4. In the GSE88808 dataset, Translation (Fig. 6B; *NES* = +2.31) and rRNA Processing (Fig. 6C; *NES* = +2.27) were enriched in the tumor group, whereas Muscle Contractions (Fig. 6D; *NES* = -2.18) and Hypertrophic Cardiomyopathy Hcm (Fig. 6E; *NES* = -2.23) were enriched in the control group. Of note, translation-related pathways showed opposite enrichment directions in TCGA-PRAD (enriched in controls) and GSE88808 (enriched in tumors), which is addressed further in the Discussion.

### 3.6 Gene Set Variation Analysis

**Figure 7:**
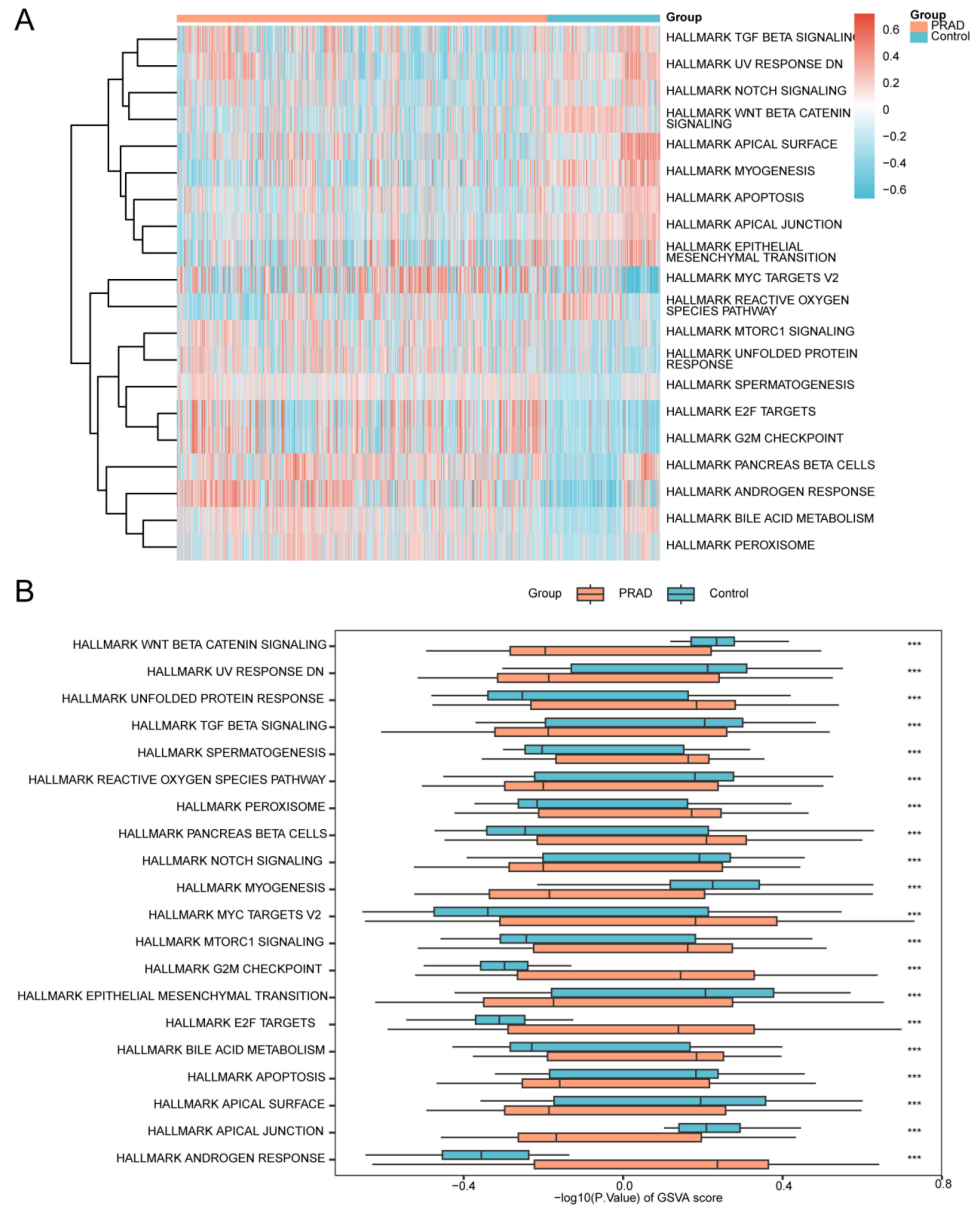
** GSVA of the TCGA-PRAD dataset.** (A) Heatmap and (B) grouped comparison plot of Gene Set Variation Analysis (GSVA) results between prostate adenocarcinoma (PRAD) and control groups in the merged prostate dataset (GTEx_TCGA-PRAD). GSVA, Gene Set Variation Analysis. ns, not significant (*p*-value ≥ 0.05); *, *p*-value < 0.05; **, *p*-value < 0.01; ***, *p*-value < 0.001. Orange represents the PRAD group; blue represents the control group. Gene sets with a *p*-value < 0.05 were considered statistically significant. In the heatmap, blue indicates low enrichment and red indicates high enrichment.

To investigate the differential activity of the h.all.v7.4.symbols.gmt gene set between the prostate cancer and control groups in the merged GTEx_TCGA-PRAD dataset, GSVA was performed on all genes across all samples. The complete results are provided in Table 5. The top 20 pathways meeting the criteria of *p*-value < 0.05 and largest absolute logFC values were selected and visualized using a heatmap to display their expression patterns between prostate cancer and control groups (Fig. 7A).

Subsequently, the Mann-Whitney *U* test was applied to validate the significance of differences, with results presented in a grouped comparison plot (Fig. 7B). The GSVA results demonstrated that the following pathways showed statistically significant differences (*p* < 0.05) between PRAD and control groups in the merged dataset:

Androgen Response, Apical Junction, Apical Surface, Apoptosis, Bile Acid Metabolism, E2f Targets, Epithelial Mesenchymal Transition, G2M Checkpoint, Mtorc1 Signaling, Myc Targets V2, Myogenesis, Notch Signaling, Pancreas Beta Cells, Peroxisome, Reactive Oxygen Species Pathway, Spermatogenesis, TGF Beta Signaling, Unfolded Protein Response, UV Response DN, and Wnt Beta Catenin Signaling.

**Figure 8:**
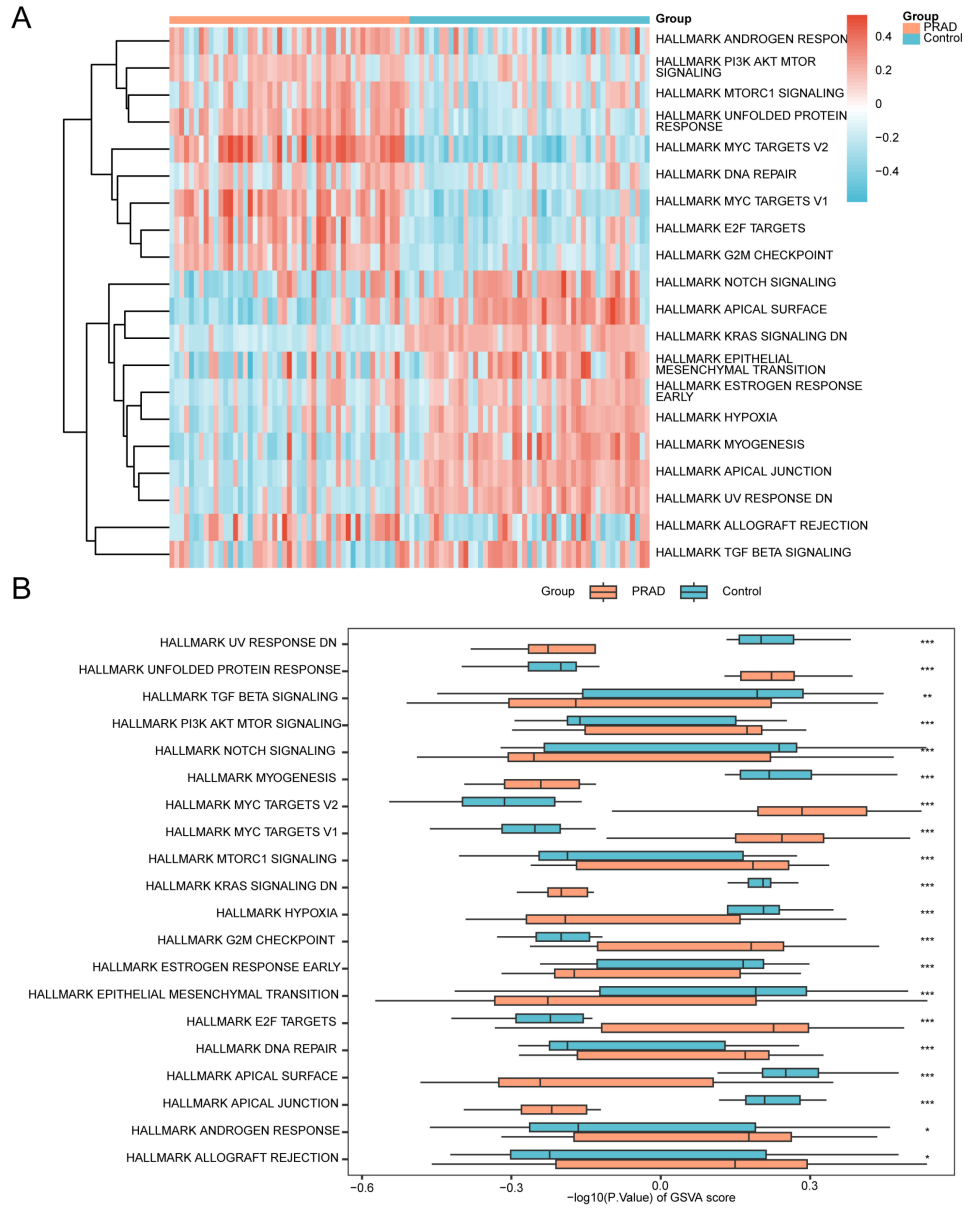
** Gene Set Variation Analysis (GSVA) of the GSE88808 dataset.** (A) Heatmap and (B) grouped comparison plot of GSVA results between prostate cancer and control groups in the GSE88808 dataset. GSVA, Gene Set Variation Analysis. ns, not significant (*p*-value ≥ 0.05); *, *p*-value < 0.05; **, *p*-value < 0.01; ***, *p*-value < 0.001. Orange represents the prostate cancer group; blue represents the control group. Gene sets with a *p*-value < 0.05 were considered statistically significant. In the heatmap, blue indicates low enrichment and red indicates high enrichment.

To investigate the differential activity of the h.all.v7.4.symbols.gmt gene set between prostate cancer and control groups in the GSE88808 dataset, GSVA was performed on all genes across all samples. Detailed results are provided in Table 6. The top 20 pathways ranked by absolute logFC values with p-value < 0.05 were selected, and their differential enrichment between the PRAD and control groups was visualized using a heatmap (Fig. 8A).

Subsequently, the Mann-Whitney U test was applied to validate these differences, with results displayed in a grouped comparison plot (Fig. 8B). The GSVA results indicated that the following pathways exhibited statistically significant differences (*p*-value < 0.05) between PRAD and control groups in the GSE88808 dataset:

Allograft Rejection, Androgen Response, Apical Junction, Apical Surface, DNA Repair, E2F Targets, Epithelial Mesenchymal Transition, Estrogen Response Early, G2M Checkpoint, Hypoxia, KRAS Signaling DN, MTORC1 Signaling, MYC Targets V1, MYC Targets V2, Myogenesis, Notch Signaling, PI3K AKT MTOR Signaling, TGF Beta Signaling, Unfolded Protein Response, and UV Response DN.

### 3.7 Construction of a Prostate Cancer Diagnostic Model

**Figure 9:**
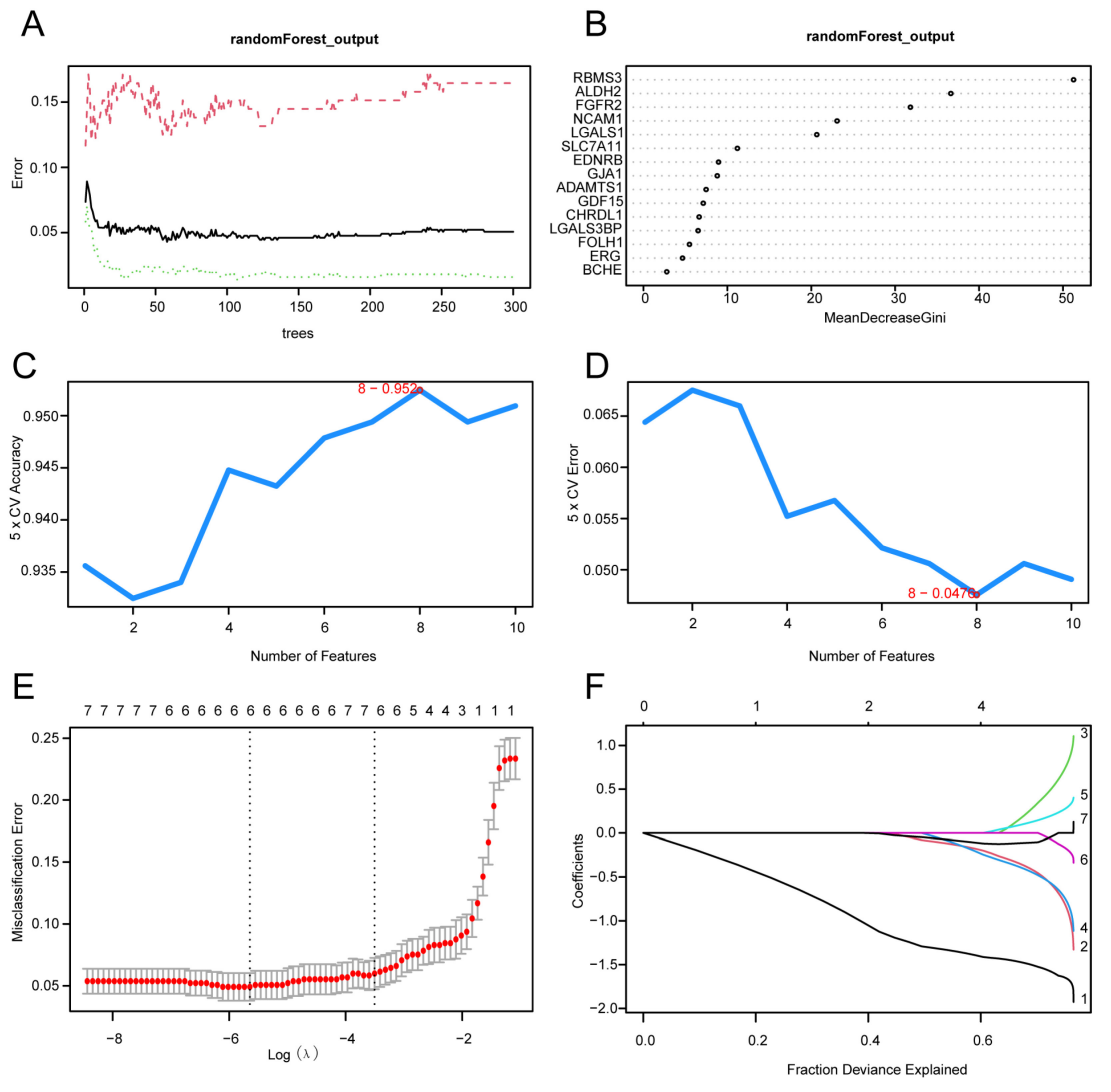
** Construction of the prostate cancer diagnostic model.** (A) Model training error plot of the Random Forest (RF) algorithm. (B) MeanDecreaseGini scatter plot of the model genes (ranked in descending order of MeanDecreaseGini). (C-D) Visualization of the number of genes yielding the highest accuracy (C) and the lowest error rate (D) from the Support Vector Machine (SVM) algorithm. (E-F) Diagnostic model plot (E) and variable trajectory plot (F) of the Least Absolute Shrinkage and Selection Operator (LASSO) regression model. SVM, Support Vector Machine; LASSO, Least Absolute Shrinkage and Selection Operator; RF, Random Forest. PRAD, Prostate adenocarcinoma; HIERDEGs, Hypoxia and Immune Escape-Related Differentially Expressed Genes.

To construct the diagnostic model, the 15 HIERDEGs were first subjected to the Random Forest (RF) algorithm for initial feature ranking. The top 10 genes with the highest importance scores were retained for subsequent selection steps. The process was visualized using a decision tree error curve (Fig. 9A) and a MeanDecreaseGini scatter plot (Fig. 9B).

Subsequently, the Support Vector Machine Recursive Feature Elimination (SVM-RFE) algorithm was employed on the 10 genes retained from the RF step, with 5-fold cross-validation, to calculate the average importance ranking (Average Rank) of each gene. The number of genes yielding the highest model accuracy (Fig. 9C) and the lowest error rate (Fig. 9D) was identified. The results indicated that the SVM model achieved optimal performance when including eight genes. Therefore, the top eight genes based on average importance ranking were selected for subsequent analysis.

Finally, a Least Absolute Shrinkage and Selection Operator (LASSO) regression analysis was performed using the eight HIERDEGs identified by the SVM model to construct a prostate cancer diagnostic model. The LASSO regression model diagram (Fig. 9E) and variable trajectory plot (Fig. 9F) were generated for visualization. The LASSO analysis identified six HIERDEGs as key genes for subsequent studies: *RBMS3*, *ALDH2*, *SLC7A11*, *FGFR2*, *GDF15*, and *NCAM1*.

### 3.8 Validation of the Prostate Cancer Diagnostic Model

**Figure 10:**
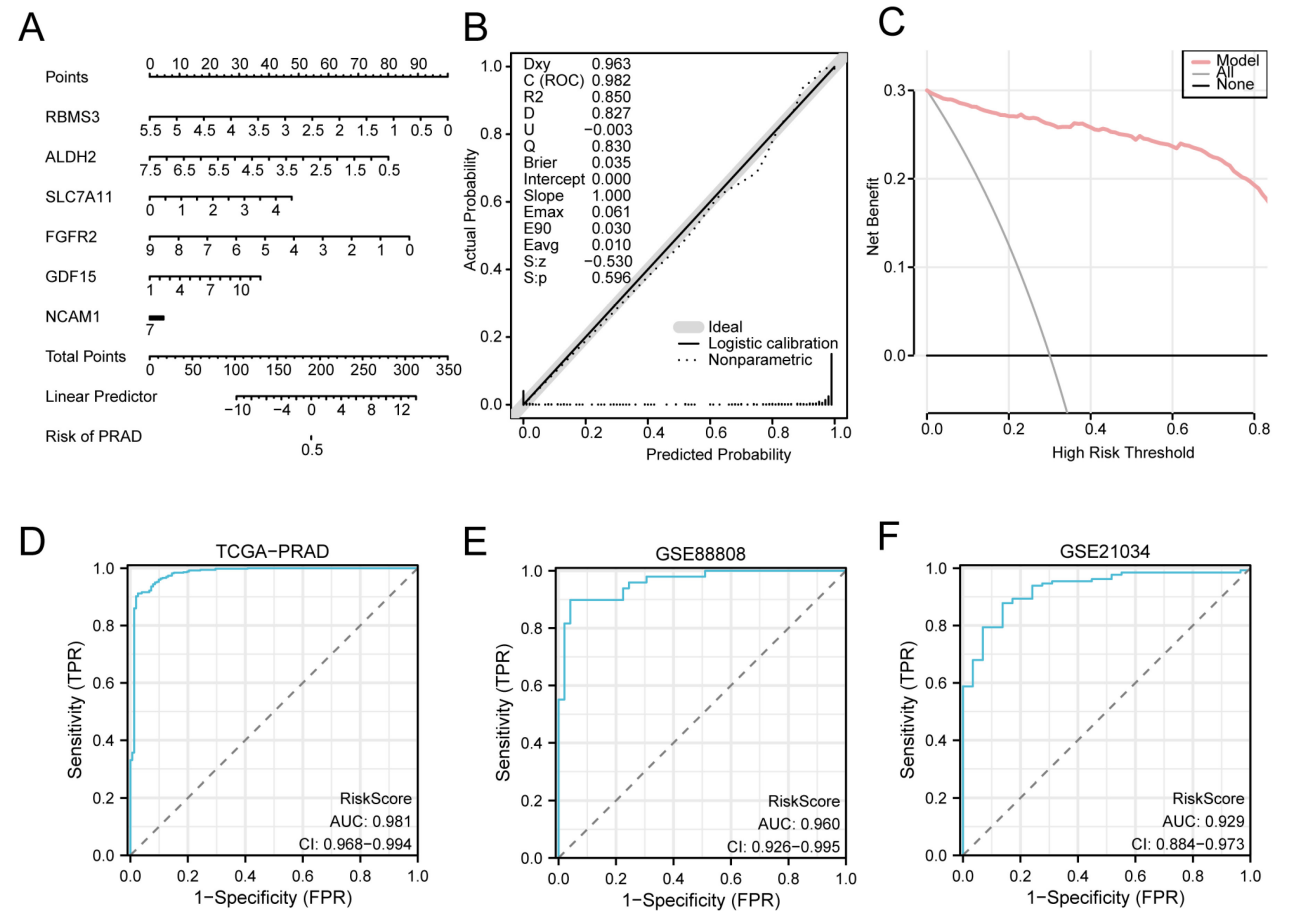
** Validation of the prostate cancer diagnostic model.** (A) Nomogram of the key genes in the diagnostic model for prostate adenocarcinoma (PRAD) within the merged prostate dataset (GTEx_TCGA-PRAD). (B-C) Calibration curve (B) and decision curve analysis (DCA) plot (C) of the PRAD diagnostic model based on the key genes in the merged dataset. (D-F) Receiver operating characteristic (ROC) curves of the RiskScore in the merged prostate dataset (GTEx_TCGA-PRAD), GSE88808, and GSE21034. PRAD, Prostate adenocarcinoma; DCA, Decision Curve Analysis; ROC, Receiver Operating Characteristic; AUC, Area Under the Curve; TPR, True Positive Rate; FPR, False Positive Rate. An AUC value > 0.5 indicates that the molecular expression shows a trend towards promoting the event. An AUC value closer to 1 indicates better diagnostic performance. An AUC above 0.9 represents high accuracy.

To further evaluate the clinical utility of the prostate cancer diagnostic model, a nomogram was constructed based on the key genes to illustrate their interrelationships within the merged GTEx_TCGA-PRAD dataset (Fig. 10A). The results indicated that the expression level of *FGFR2* contributed more substantially to the diagnostic model compared to the other variables.

Subsequently, a calibration curve was generated to assess the accuracy and discriminative ability of the diagnostic model by comparing the predicted probabilities against actual outcomes under various scenarios (Fig. 10B). DCA was further applied to evaluate the clinical net benefit of the model using the key genes in the merged dataset (Fig. 10C). The DCA results demonstrated that within a certain threshold probability range, the model's curve was consistently higher than both the “all positive” and “all negative” lines, indicating a favorable net benefit and robust clinical utility.

Additionally, ROC curves were plotted using the R package pROC based on the RiskScore derived from the merged dataset (GTEx_TCGA-PRAD) and the validation sets GSE88808 and GSE21034 (Fig. 10D, E and F). The ROC analysis revealed that the RiskScore exhibited high predictive accuracy across all datasets, with AUC values exceeding 0.9.

The RiskScore was calculated using the following formula:



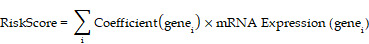



### 3.9 Validation of Expression Differences in Key Genes

**Figure 11:**
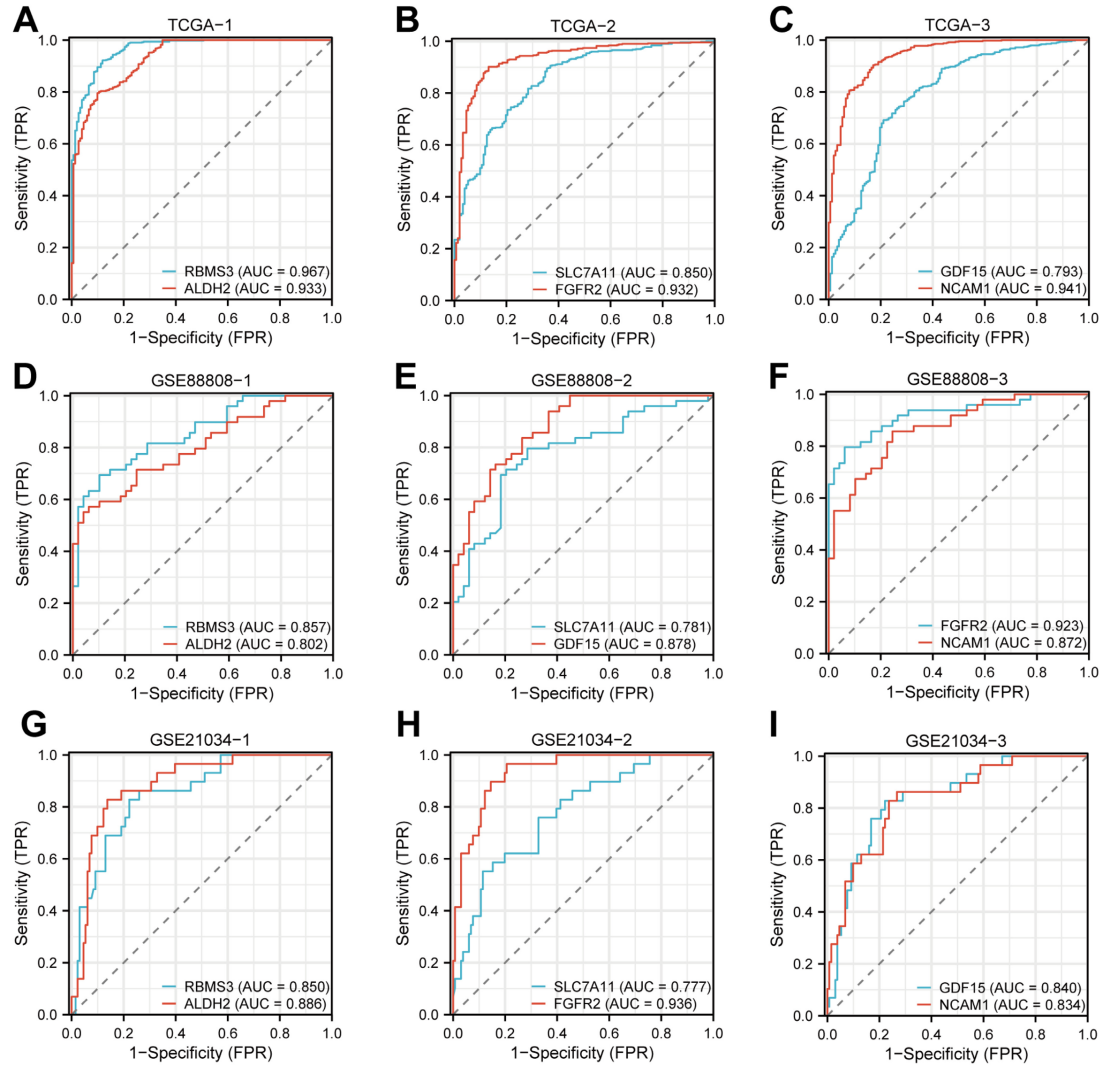
** Validation of expression differences for Key Genes.** (A-C) ROC curves for *RBMS3* (A), *ALDH2* (A), *SLC7A11* (B), *FGFR2* (B), *GDF15* (C), and *NCAM1* (C) in the merged prostate dataset (GTEx_TCGA-PRAD). (D-F) ROC curves for *RBMS3* (D), *ALDH2* (D), *SLC7A11* (E), *FGFR2* (E), *GDF15* (F), and *NCAM1* (F) in the GSE88808 dataset. (G-I) ROC curves for *RBMS3* (G), *ALDH2* (G), *SLC7A11* (H), *FGFR2* (H), *GDF15* (I), and *NCAM1* (I) in the GSE21034 dataset. An AUC value > 0.5 indicates that the molecular expression shows a trend towards promoting the event. A value closer to 1 indicates better diagnostic performance. An AUC between 0.7 and 0.9 indicates moderate accuracy, and an AUC above 0.9 represents high accuracy. ROC, Receiver Operating Characteristic; AUC, Area Under the Curve. TPR, True Positive Rate; FPR, False Positive Rate; PRAD, Prostate adenocarcinoma.

ROC curves were generated based on the expression levels of the Key Genes in the merged GTEx_TCGA-PRAD dataset using the R package pROC. As shown in Fig. 11A-C, the genes *RBMS3*, *ALDH2*, *FGFR2*, and *NCAM1* exhibited high classification accuracy (AUC > 0.9) in distinguishing between the prostate cancer and control groups, while *SLC7A11* and *GDF15* showed moderate accuracy (0.7 < AUC < 0.9).

Similarly, ROC analysis was performed using the expression levels of the Key Genes in the GSE88808 dataset. The results (Fig. 11D-F) indicated that FGFR2 achieved high accuracy (AUC > 0.9), whereas *RBMS3*, *ALDH2*, *SLC7A11*, and *GDF15* demonstrated moderate accuracy (0.7 < AUC < 0.9) in classifying prostate cancer versus control samples. Finally, ROC curves based on the GSE21034 dataset (Fig. 11G-I) revealed that all Key Genes—*RBMS3*, *ALDH2*, *SLC7A11*, *FGFR2*, *GDF15*, and *NCAM1*—showed moderate accuracy (0.7 < AUC < 0.9) in differentiating between the prostate cancer and control groups.

### 3.10 Gene Set Enrichment Analysis in High- and Low-Score Groups

To characterize molecular features associated with the diagnostic model across prostate cancer samples, we stratified the tumor specimens by the median RiskScore of the same model. The following GSEA, GSVA, and immune infiltration analyses are exploratory in nature and aim to describe pathway- and immune- related patterns linked to this gene signature, rather than to independently validate biological mechanisms. For these exploratory analyses, differential expression analysis between the model-defined groups was first performed using the R package limma. A total of 1,247 differentially expressed genes (DEGs) were identified under the threshold of |logFC| > 1 and *p*-value < 0.05. Among these, 78 genes were up-regulated (logFC > 1 and *p*-value < 0.05), and 1169 genes were down-regulated (logFC < -1 and *p*-value < 0.05). The results are visualized in a volcano plot (Figure S1).

**Figure 12:**
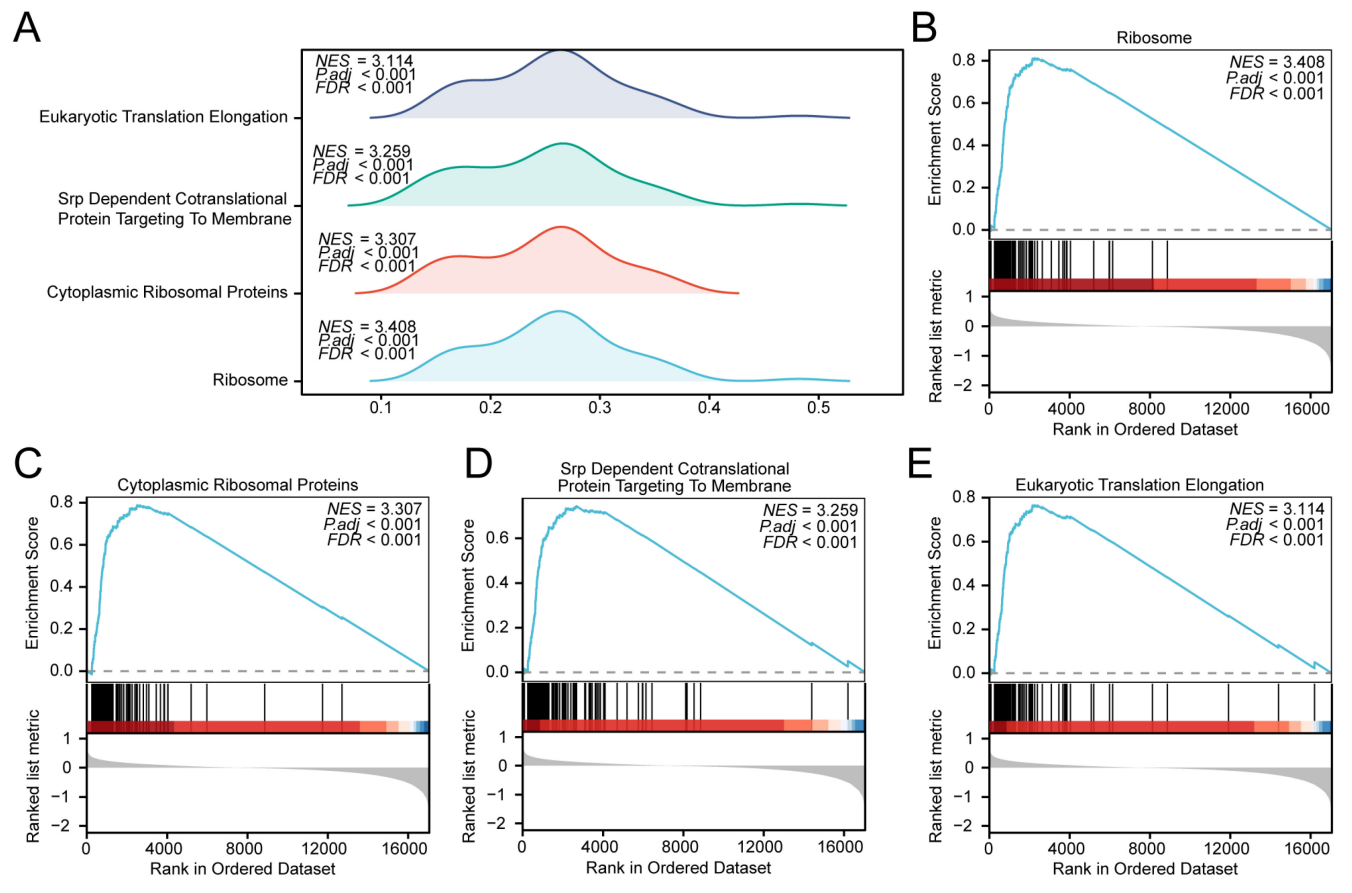
** Gene Set Enrichment Analysis (GSEA) in high- and low-score groups based on the GTEx_TCGA-PRAD dataset.** (A) Mountain plot from the GSEA showing four significantly enriched biological functions in the prostate cancer samples of the GTEx_TCGA-PRAD dataset. (B-E) GSEA plots demonstrating significant enrichment of all genes in RIBOSOME (B), Cytoplasmic Ribosomal Proteins (C), SRP Dependent Cotranslational Protein Targeting To Membrane (D), and Eukaryotic Translation Elongation (E). GSEA, Gene Set Enrichment Analysis. Significantly enriched gene sets were selected using an adjusted *p*-value (adj. *p*) < 0.05 and a false discovery rate (FDR, q-value) < 0.25, with p-value adjustment performed using the Benjamini-Hochberg (BH) method. Note: Groups were defined by the median RiskScore of the diagnostic model; therefore, the observed differences are exploratory and reflect heterogeneity relative to the model.

To assess the functional implications of global gene expression patterns related to prostate cancer pathogenesis in the high- and low-score groups, Gene Set Enrichment Analysis (GSEA) was conducted based on the logFC values of all genes between the high- and low-score groups within the prostate cancer samples of the merged dataset. The functional associations between gene expression and biological processes, cellular components, and molecular functions are illustrated in a mountain plot (Fig. 12A), with detailed results provided in Table 7. In these model-based comparisons, several pathways showed differential enrichment, including RIBOSOME (Fig. 12B), Cytoplasmic Ribosomal Proteins (Fig. 12C), SRP Dependent Cotranslational Protein Targeting To Membrane (Fig. 12D), and Eukaryotic Translation Elongation (Fig. 12E).

### 3.11 Gene Set Variation Analysis in High- and Low-Score Groups

**Figure 13:**
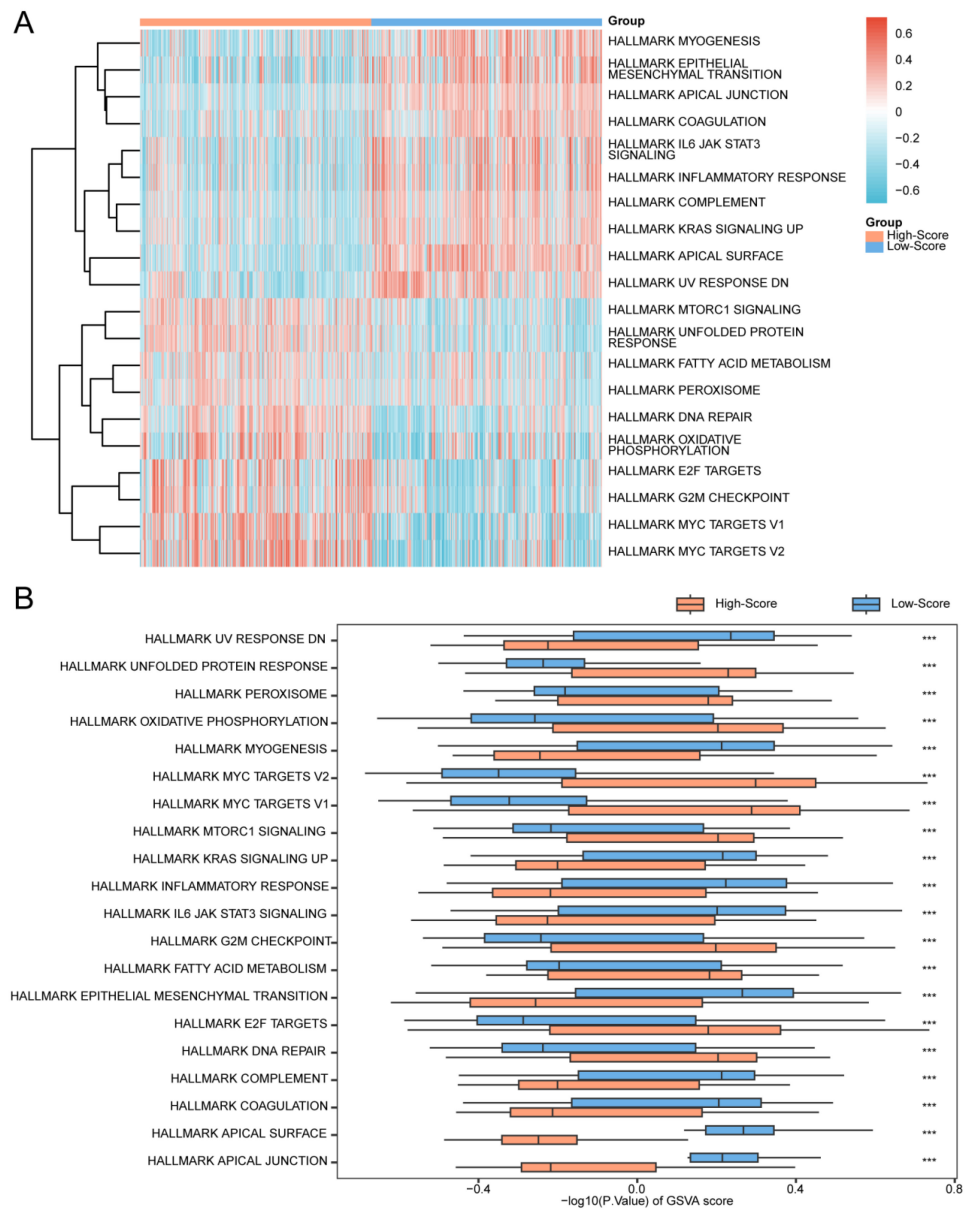
** Gene Set Variation Analysis (GSVA) in high- and low-score prostate cancer groups.** (A) Heatmap and (B) grouped comparison plot of GSVA results between high- and low-score groups within the prostate cancer samples of the merged GTEx_TCGA-PRAD dataset. GSVA, Gene Set Variation Analysis. ns, not significant (*p*-value ≥ 0.05); *, *p*-value < 0.05; **, *p*-value < 0.01; ***, *p*-value < 0.001. Orange represents the high-score group; dark blue represents the low-score group. Gene sets with a *p*-value < 0.05 were considered statistically significant. In the heatmap, blue indicates low enrichment and red indicates high enrichment. Note: Groups were defined by the median RiskScore of the diagnostic model; therefore, the observed differences are exploratory and reflect heterogeneity relative to the model.

To further characterize pathway activity patterns associated with the model-defined groups, GSVA was performed on the same prostate cancer samples of the merged GTEx_TCGA-PRAD dataset using the h.all.v7.4.symbols.gmt gene set. These analyses are descriptive and serve to illustrate heterogeneity relative to the gene signature. Detailed results are provided in Table 8. The top 20 pathways ranked by absolute logFC values with a *p*-value < 0.05 were selected, and their enrichment patterns between the high- and low-score groups were visualized using a heatmap (Fig. 13A).

Subsequently, the Mann-Whitney *U* test was applied to validate the significance of the observed differences, with results illustrated in a grouped comparison plot (Fig. 13B). The GSVA results demonstrated that the following pathways were statistically significant (*p*-value < 0.05) between the high- and low-score groups:

Apical Junction, Apical Surface, Coagulation, Complement, DNA Repair, E2F Targets, Epithelial Mesenchymal Transition, Fatty Acid Metabolism, G2M Checkpoint, IL6-JAK-STAT3 Signaling, Inflammatory Response, KRAS Signaling Up, MTORC1 Signaling, MYC Targets V1, MYC Targets V2, Myogenesis, Oxidative Phosphorylation, Peroxisome, Unfolded Protein Response, And UV Response DN.

### 3.12 Immune Infiltration Analysis in High- and Low-Score Groups

**Figure 14:**
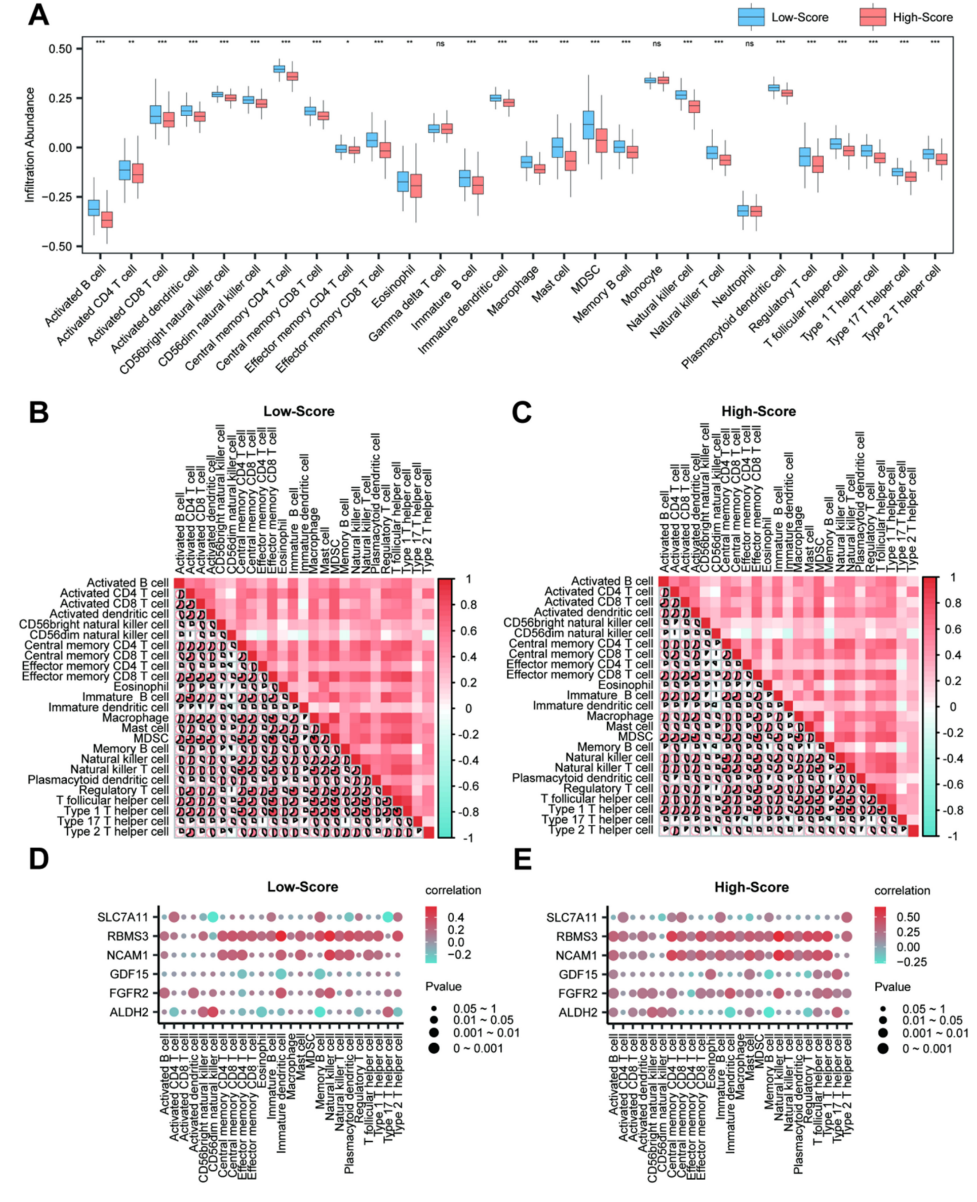
** Immune infiltration analysis by ssGSEA algorithm.** (A) Grouped comparison plot of immune cell infiltration between high- and low-score groups in prostate cancer samples. (B-C) Correlation heatmaps of immune cell infiltration abundance in the high-score (B) and low-score (C) groups of samples. (D-E) Bubble plots showing correlations between immune cell infiltration abundance and key genes in the high-score (D) and low-score (E) groups of prostate adenocarcinoma (PRAD) samples. ssGSEA, single-sample Gene-Set Enrichment Analysis. ns, not significant (*p*-value ≥ 0.05); *, *p*-value < 0.05; **, *p*-value < 0.01; ***, *p*-value < 0.001. Absolute correlation coefficient (*r* value) < 0.3 indicates weak or no correlation; 0.3-0.5 indicates weak correlation; 0.5-0.8 indicates moderate correlation; > 0.8 indicates strong correlation. Orange represents the high-score group; dark blue represents the low-score group. Red indicates positive correlation; blue indicates negative correlation. Color intensity represents the strength of correlation. Note: Groups were defined by the median RiskScore of the diagnostic model; therefore, the observed differences are exploratory and reflect heterogeneity relative to the model.

To describe immune infiltration patterns across the model-derived groups, the relative abundance of 28 immune cell types was quantified in the high- and low-score groups using the ssGSEA algorithm. A grouped comparison plot illustrated the differential infiltration levels of immune cells between the two groups (Fig. 14A). The results indicated that 25 immune cell types showed statistically significant differences (*p*-value < 0.05): Activated B cell, Activated CD4+ T cell, Activated CD8+ T cell, Activated dendritic cell, CD56bright natural killer cell, CD56dim natural killer cell, Central memory CD4+ T cell, Central memory CD8+ T cell, Effector memory CD4+ T cell, Effector memory CD8+ T cell, Eosinophil, Immature B cell, Immature dendritic cell, Macrophage, Mast cell, MDSC, Memory B cell, Natural killer cell, Natural killer T cell, Plasmacytoid dendritic cell, Regulatory T cell, T follicular helper cell, Type 1 T helper cell, Type 17 T helper cell, and Type 2 T helper cell.

Subsequently, correlation heatmaps were generated to visualize the relationships among the 25 immune cell types in both high- and low-score groups (Fig. 14B and C). The analysis revealed that most immune cells exhibited notable positive correlations in both groups. However, the high-score group contained more immune cells with negative correlations.

Finally, bubble plots were used to display the correlations between the key genes and immune cell infiltration levels (Fig. 14D and E). In these descriptive comparisons, positive correlations were observed between key genes and certain immune cell types in both score groups. For instance, *RBMS3* showed correlations with Natural killer cells (*r* = 0.667, *p* < 0.05) and dendritic cells (*r* = 0.572, *p* < 0.05) within the model-defined groups; whether this reflects a role in anti-tumor immunity requires further investigation.

### 3.13 Protein-protein Interaction Network and Regulatory Network Analysis of Key Genes

**Figure 15:**
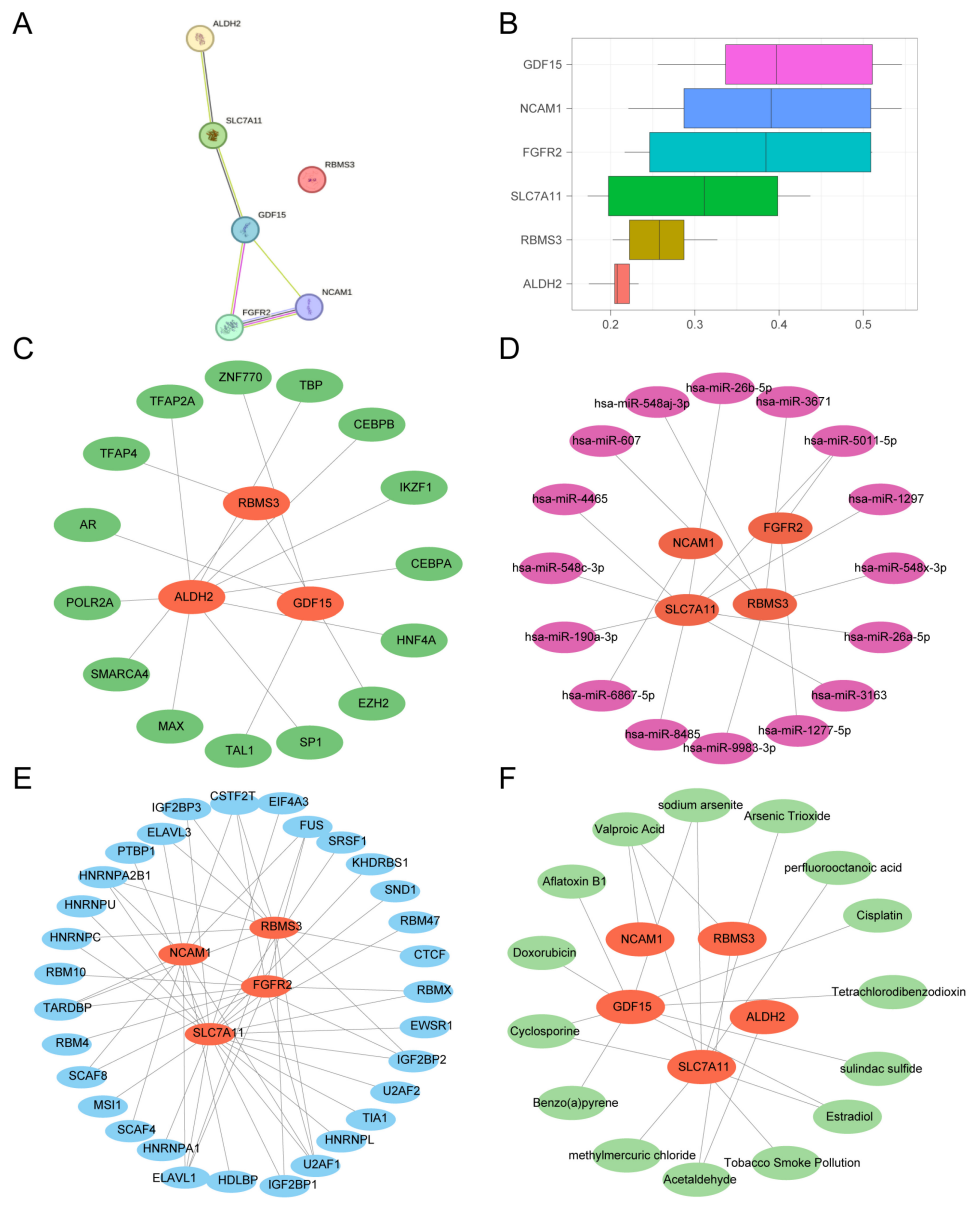
** Protein-protein interaction (PPI) and regulatory network analysis of Key Genes.** (A) PPI network of key genes calculated by the STRING database. (B) Box plot of functional similarity (Friends) analysis results for key genes. (C) mRNA-transcription factor (TF) regulatory network of key genes. (D) mRNA-miRNA regulatory network of key genes. (E) mRNA-RNA binding protein (RBP) regulatory network of key genes. (F) mRNA-drug regulatory network of key genes. TF, Transcription Factor; RBP, RNA-Binding Protein. Orange represents mRNA; dark green represents TF; pink represents miRNA; blue represents RBP; light green represents drug.

First, a PPI network was constructed based on the six key genes using the STRING database (Fig. 15A). Functional similarity (Friends) analysis was performed to evaluate the biological relevance of these genes in prostate cancer (Fig. 15B). The results indicated that *GDF15* might play a crucial role in prostate cancer, as it was the gene closest to the pre-defined cut-off value (0.60).

Next, TFs predicted to bind the key genes were retrieved from the ChIPBase database. An mRNA-TF regulatory network was constructed and visualized using Cytoscape (Fig. 15C), comprising 3 key genes and 15 TFs (see Table S3 for details).

Subsequently, miRNAs associated with the key genes were obtained from the StarBase database. An mRNA-miRNA regulatory network was built and visualized (Fig. 15D), consisting of 4 key genes and 16 miRNAs (Table S4).

Then, RNA-binding proteins (RBPs) targeting the key genes were predicted using StarBase. The resulting mRNA-RBP regulatory network (Fig. 15E) included 4 key genes and 41 RBPs (Table S5).

Finally, the CTD was used to identify potential drugs or molecular compounds associated with the key genes. An mRNA-drug interaction network was constructed and visualized (Fig. 15F), involving 5 key genes and 15 drugs/compounds (Table S6).

## 4. Discussion

Prostate cancer is a prevalent malignancy among men worldwide, with tumor progression often influenced by hypoxia and immune escape mechanisms within the tumor microenvironment. Hypoxia can drive gene expression changes that foster an immunosuppressive landscape, as evidenced in lung adenocarcinoma, where *ERO1L* overexpression under hypoxic conditions recruits immunosuppressive cells like regulatory T cells and M2 macrophages, correlating with poor prognosis and therapy resistance 30. Similarly, in cancers such as follicular lymphoma, *GLUT1* upregulation promotes an immunosuppressive niche through M2 macrophages and Tregs, linked to early disease progression 31. Current research has advanced in identifying hypoxia and immune-related gene signatures in various malignancies, including the role of factors like IgG4 in mediating M2 polarization in esophageal cancer 32 and the impact of metabolic genes on immune cell dynamics in colorectal cancer 33. However, a significant gap remains in prostate cancer, where integrated bioinformatic approaches combining hypoxia and immune escape elements are limited, hindering the development of robust diagnostic models for molecular characterization. This underscores the need for comprehensive studies to bridge this gap and enhance clinical applications.

In this study, we systematically identified and validated a hypoxia and immune escape-related gene signature for prostate cancer through integrative bioinformatic analysis. Our findings highlight the critical roles of these molecular features in prostate carcinogenesis and offer a novel diagnostic model with potential clinical utility.

We identified 15 HIERDEGs across three independent datasets, with six key genes—*RBMS3*, *ALDH2*, *SLC7A11*, *FGFR2*, *GDF15*, and *NCAM1*—consistently contributing to prostate cancer diagnosis. Among these, *GDF15* has been previously implicated in promoting tumor growth and metastasis in various cancers, including prostate adenocarcinoma, through enhancing cell invasion and resistance to therapy 34-36. Hypoxia, a hallmark of solid tumors, drives intratumoral heterogeneity and immune evasion by activating hypoxia-inducible factors (HIFs), which orchestrate metabolic reprogramming and suppress antitumor immunity 37. Specifically, hypoxia-mediated HIF-1α upregulation promotes immune escape by downregulating major histocompatibility complex class I chain-related genes (e.g., MIC), facilitating soluble MIC release and impairing natural killer cell cytotoxicity 38. Additionally, hypoxia-induced exosomal *circHIF1A* from cancer-associated fibroblasts enhances PD-L1 expression via HuR binding, further suppressing T-cell activity and promoting immune evasion 39. Hypoxia also strengthens the* CD47-SIRPα* axis through ZEB1 activation, enabling cancer cells to evade phagocytosis by tumor-associated macrophages 40. Metabolic shifts, such as aerobic glycolysis and lactate acidification in hypoxic microenvironments, inhibit immune cell function and foster a suppressive tumor immune microenvironment 41. Furthermore, hypoxia-induced *GAL3ST1*-sulfatide accumulation enhances tumor cell-platelet binding, protecting cancer cells from natural killer cell-mediated cytotoxicity 42. These mechanisms collectively underscore the critical role of hypoxia in driving immune evasion, highlighting the potential of targeting hypoxia-related pathways to improve diagnostic and therapeutic strategies for prostate cancer 43.

GO enrichment analysis of the 15 HIERDEGs revealed their involvement in oligopeptide transmembrane transport and smooth muscle cell proliferation (Table 2). Separately, GSEA of genome-wide expression profiles identified translation-related pathways as differentially enriched between tumor and control groups, although the enrichment direction was not uniform across datasets. These biological processes have been associated with prostate cancer progression and metabolic adaptation under hypoxia 44. In the TCGA-PRAD dataset, translation-related pathways (e.g., Eukaryotic Translation Initiation and Elongation) were enriched in the control group rather than in tumor samples. In contrast, the GSE88808 dataset showed enrichment of these pathways in the tumor group. This directional inconsistency across cohorts suggests that the relationship between translation-related pathway activity and prostate cancer may be context-dependent, and these results should be interpreted with consideration of cohort-specific characteristics rather than generalized to a uniform tumor-promoting role 45. These findings indicate that translation-related processes are differentially represented across prostate cancer datasets, though the direction of alteration was not consistent. Further investigation is needed to clarify the role of translational dysregulation in prostate cancer and to determine whether these pathways have potential therapeutic relevance. Beyond translation-related pathways, the GO analysis highlighted several other biological processes potentially relevant to prostate cancer, such as oligopeptide transport and smooth muscle proliferation. Recent studies have further elucidated the mechanisms linking oligopeptide transmembrane transport and smooth muscle proliferation to tumor microenvironment adaptation in prostate cancer. For example, PSMA-mediated oligopeptide transport facilitates nutrient uptake under hypoxic conditions, supporting metabolic adaptation and immune evasion in metastatic castration-resistant prostate cancer 46. Additionally, cancer stem cells drive smooth muscle proliferation through regulatory pathways such as TGF-β signaling, which interacts with the tumor microenvironment to promote therapy resistance and progression 47. Neural innervation within the microenvironment also modulates these processes, contributing to aggressive phenotypes and adaptation in neuroendocrine prostate cancer 48. The directional discrepancy between TCGA-PRAD and GSE88808 for translation-related pathways may reflect differences in sample composition, platform characteristics, or tumor heterogeneity between cohorts. This observation highlights the importance of evaluating pathway enrichment across multiple independent datasets before drawing biological conclusions.

Using a machine learning-based approach incorporating Random Forest, SVM-RFE, and LASSO regression, we developed a six-gene diagnostic signature that demonstrated consistently high accuracy (AUC > 0.9) across all validation sets. The robust performance of the model in both internal and external cohorts highlights its favorable generalizability and suggests applicability across diverse clinical and platform settings. Whether this gene signature can provide additional value beyond existing clinical parameters such as PSA levels and Gleason scores remains to be evaluated in future studies with complete clinical annotation and prospective designs.

Regulatory network analyses revealed intricate interactions between key genes and transcriptional regulators, miRNAs, RNA-binding proteins, and druggable compounds. For instance, several predicted transcription factors and miRNAs have previously been associated with prostate cancer pathogenesis, supporting the biological plausibility of our findings 49, 50. The drug-gene interaction analysis highlighted compounds such as Metformin and Curcumin, which have known anti-tumor properties, suggesting potential repositioning opportunities for prostate cancer treatment. These insights pave the way for hypothesis-driven experimental studies to validate candidate therapeutic targets.

It should be noted that the high- and low-score groups were defined based on the median RiskScore derived from the diagnostic model, and this grouping was intended to explore molecular heterogeneity within prostate cancer samples rather than to predict clinical prognosis. Furthermore, immune infiltration patterns differed between the model-defined high- and low-score groups. The high-score group exhibited higher infiltration of immunosuppressive cells such as M2 macrophages and regulatory T cells, a pattern that, in other contexts, has been associated with poor prognosis. However, because the grouping was derived from the same diagnostic model, these observations should be viewed as descriptive and hypothesis-generating rather than as independent prognostic evidence 51. For instance, in lung adenocarcinoma, ERO1L overexpression was linked to the recruitment of Tregs and M2 macrophages, fostering an immunosuppressive tumor microenvironment that conferred resistance to immunotherapy and correlated with adverse outcomes 30. Similarly, in follicular lymphoma, *GLUT1* overexpression promoted an immunosuppressive niche by inducing M2 macrophages and Tregs, which was directly related to disease progression within 24 months (POD24), highlighting their role in driving aggressive disease and treatment failure 31. Additionally, altered coordination between innate and adaptive immune responses was observed, indicative of an immune-evasive phenotype. Notably, RBMS3 showed notable correlations with NK cells and dendritic cells, implying a possible role in modulating anti-tumor immunity. These findings suggest that the proposed gene signature might also serve as an indicator of immune contexture, thereby informing immunotherapeutic strategies; for example, in breast and liver cancers, reducing M2 macrophages and Tregs has been demonstrated to enhance immunotherapy efficacy and remodel the tumor microenvironment toward a more immunoreactive state 52.

Several limitations should be acknowledged. First, although we integrated multiple public cohorts to enhance reproducibility, inherent differences in sample processing across platforms might still affect gene expression measurements. It should be noted that TCGA adjacent-normal tissues and GTEx normal prostate samples differ in terms of sample collection and processing workflows. In this study, these datasets were analyzed after harmonizing expression units and gene annotation, and therefore findings derived from the merged analyses should be interpreted with an awareness of the different data sources. Such differences may contribute to the observed asymmetry in the number of up- and down-regulated genes in the differential expression analysis. Nevertheless, the consistent performance of the model in independent GEO datasets, which were analyzed separately without merging, suggests that the overall findings are robust and reproducible. Additionally, it should be noted that keyword-based retrieval from the GeneCards database may include genes with only weak associations with hypoxia or immune escape. Although we employed multi-step strategies including literature cross-validation, differential expression analysis, and machine learning-based feature selection to minimize such non-specific effects, potential background noise may still exist. Nevertheless, the consistent performance of the final six-gene model across multiple independent datasets suggests its biological relevance and diagnostic robustness. Second, although the model demonstrated robust predictive performance across independent validation datasets, indicating satisfactory accuracy and reproducibility at the data level, the study relies solely on computational and bioinformatic evidence. Due to the reliance on public databases and the lack of access to clinical specimens and experimental resources, we have not yet performed additional biological validation (such as qPCR, Western blot, or immunohistochemistry) to confirm the expression patterns of the key genes at the tissue or cellular level. Functional validation through in vitro and in vivo experiments is necessary to establish causality. Third, although the gene signature developed in this study demonstrated high diagnostic accuracy across multiple cohorts, the direct association between the model and clinical parameters (such as Gleason score, tumor stage, PSA level, and survival outcomes) has not been systematically validated due to incomplete clinical information in some public datasets. This limitation, to some extent, restricts the generalizability of the model to clinical practice. Future studies incorporating multi-center clinical cohorts, follow-up data, and experimental validation are warranted to further evaluate the potential utility of this signature in risk stratification and prognostic prediction, thereby facilitating its clinical translation.

Furthermore, a key limitation is that the high- and low-score groups were defined using the median RiskScore of the diagnostic model itself. Consequently, the pathway enrichment and immune infiltration analyses based on these groups represent exploratory descriptions of molecular heterogeneity relative to the gene signature, not independent validation of biological mechanisms. This inherent circularity necessitates cautious interpretation of the biological significance of these findings.

Additionally, the diagnostic model achieved a high AUC in the training dataset, yet internal cross-validation was not incorporated into the current analysis. This raises the possibility of overfitting, and the stability and generalizability of the model should therefore be interpreted with caution. Although the model maintained robust performance in independent external validation cohorts, future studies integrating internal cross-validation or bootstrapping are warranted to further assess its reproducibility.

In conclusion, we have established a hypoxia and immune escape-related gene signature with robust diagnostic performance for prostate cancer, which offers insights into the underlying molecular mechanisms. The diagnostic model demonstrates high accuracy and robustness across datasets, holding promise for clinical application in early detection and personalized treatment planning. Future work should focus on experimental validation and integration of the model into clinical workflow for improved prostate cancer management.

## Supplementary Material

Supplementary figure and tables.

## Figures and Tables

**Table 1:** GEO and TCGA Chip Information

	GTEx_TCGA-PRAD	GSE88808	GSE21034
Platform	/	GPL22571	GPL5188
Species	Homo sapiens	Homo sapiens	Homo sapiens
Tissue	Prostate	Prostate	Prostate
Samples in PRAD group	499	49	131
Samples in Control group	152	49	29
Reference	/	PMID: 28027300	PMID: 20579941

GEO, Gene Expression Omnibus; PRAD, prostate adenocarcinoma.

**Table 2:** Results of GO Enrichment Analysis for HIERDEGs

ONTOLOGY	ID	Description	Gene Ratio	Bg Ratio	*p* value	*p*.adjust	*q* value
BP	GO:0035672	oligopeptide transmembrane transport	2/15	14/18800	5.38e-05	0.0195	BP
BP	GO:0048661	positive regulation of smooth muscle cell proliferation	3/15	95/18800	5.44e-05	0.0195	BP
BP	GO:0006857	oligopeptide transport	2/15	16/18800	7.08e-05	0.0195	BP
BP	GO:0097164	ammonium ion metabolic process	2/15	23/18800	0.0001	0.0258	BP
BP	GO:0072337	modified amino acid transport	2/15	32/18800	0.0003	0.0258	BP
CC	GO:0062023	collagen-containing extracellular matrix	6/15	429/19594	4.5e-07	2.03e-05	CC
CC	GO:0005788	endoplasmic reticulum lumen	3/15	311/19594	0.0016	0.0352	CC
CC	GO:0072562	blood microparticle	2/15	147/19594	0.0055	0.0687	CC
CC	GO:0005641	nuclear envelope lumen	1/15	10/19594	0.0076	0.0687	CC
CC	GO:0005916	fascia adherens	1/15	10/19594	0.0076	0.0687	CC
MF	GO:0072349	modified amino acid transmembrane transporter activity	2/14	22/18410	0.0001	0.0103	MF
MF	GO:1901682	sulfur compound transmembrane transporter activity	2/14	52/18410	0.0007	0.0293	MF
MF	GO:0042277	peptide binding	3/14	322/18410	0.0017	0.0468	MF
MF	GO:0033218	amide binding	3/14	402/18410	0.0031	0.0565	MF
MF	GO:0052689	carboxylic ester hydrolase activity	2/14	149/18410	0.0056	0.0565	MF

GO, Gene Ontology; BP, Biological Process; CC, Cellular Component; MF, Molecular Function; HIERDEGs, Hypoxia and Immune Escape-Related Differentially Expressed Genes.

**Table 3:** Results of GSEA for TCGA-PRAD

ID	Set Size	Enrichment Score	NES	*p* value	*p.*adjust	*q* value
REACTOME_EUKARYOTIC_TRANSLATION_INITIATION	120	-0.5996134	-1.618274	5.26e-10	1.29e-06	1.16e-06
REACTOME_SMOOTH_MUSCLE_CONTRACTION	40	-0.7372383	-1.862927	1.28e-08	1.14e-05	1.03e-05
WP_CYTOPLASMIC_RIBOSOMAL_PROTEINS	88	-0.6201439	-1.650827	2.32e-08	1.14e-05	1.03e-05
REACTOME_EUKARYOTIC_TRANSLATION_ELONGATION	93	-0.6107496	-1.629429	2.01e-08	1.14e-05	1.03e-05

GSEA, Gene Set Enrichment Analysis. NES, normalized enrichment score. PRAD, prostate adenocarcinoma.

**Table 4:** Results of GSEA for GSE88808

ID	Set Size	Enrichment Score	NES	*p* value	*p*.adjust	*q* value
REACTOME_TRANSLATION	257	0.5772615	2.307651	1e-10	8.25e-08	7.13e-08
REACTOME_RRNA_PROCESSING	180	0.5888950	2.271053	1e-10	8.25e-08	7.13e-08
REACTOME_MUSCLE_CONTRACTION	195	-0.6055767	-2.178558	1e-10	8.25e-08	7.13e-08
KEGG_HYPERTROPHIC_CARDIOMYOPATHY_HCM	81	-0.6943185	-2.230997	9.76e-10	6.04e-07	5.22e-07

GSEA, Gene Set Enrichment Analysis. NES, normalized enrichment score.

**Table 5:** Results of GSVA for TCGA-PRAD

Pathway	logFC	AveExpr	*t*	*p* value	adj.*p* value	*B*
MYOGENESIS	0.270050096	-0.011617993	10.01039719	4.43E-22	7.38E-21	39.12247028
WNT_BETA_CATENIN_SIGNALING	0.235532013	-0.014545888	9.957336725	7.06E-22	8.82E-21	38.66215581
APICAL_JUNCTION	0.213600863	0.000331185	9.703573208	6.39E-21	5.32E-20	36.48593761
UV_RESPONSE_DN	0.206413679	-0.008306277	7.829450667	1.92E-14	9.58E-14	21.78756818
EPITHELIAL_MESENCHYMAL_TRANSITION	0.165329552	-0.010436003	5.318153943	1.43E-07	3.97E-07	6.336186712
APICAL_SURFACE	0.162848351	-0.00252	6.000216719	3.22E-09	1.15E-08	10.01345262
TGF_BETA_SIGNALING	0.13179522	-0.010673685	4.676545604	3.53E-06	8.40E-06	3.250476734
NOTCH_SIGNALING	0.120700827	-0.022077846	4.786944287	2.08E-06	5.21E-06	3.755150175
REACTIVE_OXYGEN_SPECIES_PATHWAY	0.118166731	-0.034411752	4.440256455	1.05E-05	2.28E-05	2.207468366
APOPTOSIS	0.099384669	-0.007765988	4.212150598	2.88E-05	5.53E-05	1.249003186
MTORC1_SIGNALING	-0.146743441	-0.006367699	-5.77930503	1.15E-08	3.83E-08	8.778424513
PEROXISOME	-0.147066431	-0.002690951	-6.552823721	1.13E-10	4.33E-10	13.28357796
BILE_ACID_METABOLISM	-0.171693829	0.022697126	-8.00178647	5.40E-15	3.00E-14	23.03291842
SPERMATOGENESIS	-0.174387415	0.019995301	-9.53658568	2.66E-20	1.90E-19	35.07698759
UNFOLDED_PROTEIN_RESPONSE	-0.196261648	-0.008487084	-7.733939886	3.83E-14	1.74E-13	21.10709556
PANCREAS_BETA_CELLS	-0.207766185	0.043426289	-8.013559782	4.95E-15	3.00E-14	23.11881391
MYC_TARGETS_V2	-0.247840334	-0.009801235	-7.103970078	3.10E-12	1.29E-11	16.79597249
G2M_CHECKPOINT	-0.286339876	-0.030105053	-10.25570602	5.03E-23	1.26E-21	41.27395329
E2F_TARGETS	-0.291785959	-0.037980987	-9.866052649	1.57E-21	1.57E-20	37.87449414
ANDROGEN_RESPONSE	-0.357240225	0.022799337	-12.23764759	3.17E-31	1.58E-29	59.97107512

GSVA, Gene Set Variation Analysis. PRAD, prostate adenocarcinoma.

**Table 6:** Results of GSVA for GSE88808

Pathway	logFC	AveExpr	*t*	*p* value	adj.*p* value	*B*
APICAL_SURFACE	0.379902173	0.030187863	8.742337871	2.19E-14	1.83E-13	22.01802151
MYOGENESIS	0.338525885	0.002199833	7.717426348	4.75E-12	2.97E-11	16.70009857
APICAL_JUNCTION	0.336280388	0.020253887	9.741711268	1.04E-16	1.29E-15	27.3251424
KRAS_SIGNALING_DN	0.320147045	0.009952432	11.07702471	7.63E-20	1.99E-18	34.48486304
UV_RESPONSE_DN	0.316767125	0.015639036	8.545165115	6.24E-14	4.46E-13	20.98257866
EPITHELIAL_MESENCHYMAL_TRANSITION	0.234592934	0.016210693	4.524449725	1.48E-05	6.18E-05	2.072221889
HYPOXIA	0.219944184	0.013667596	5.232889657	7.60E-07	3.45E-06	4.945419051
NOTCH_SIGNALING	0.203902311	-0.006093818	3.789649608	0.00024184	0.000755751	-0.59248945
ESTROGEN_RESPONSE_EARLY	0.168972569	0.009278901	4.344669613	3.02E-05	0.000116323	1.388563825
TGF_BETA_SIGNALING	0.142029131	0.016729953	2.674283782	0.00858042	0.018653088	-3.904845178
ANDROGEN_RESPONSE	-0.128322153	-0.000662342	-2.543942416	0.012288218	0.025600453	-4.227407849
ALLOGRAFT_REJECTION	-0.134423279	-0.013299146	-2.366215488	0.019644222	0.039288444	-4.643939302
DNA_REPAIR	-0.154309229	-0.02276905	-3.749769885	0.000278763	0.000819892	-0.726880307
PI3K_AKT_MTOR_SIGNALING	-0.157724094	0.006879271	-4.246282799	4.43E-05	0.000154931	1.02283246
MTORC1_SIGNALING	-0.180064039	0.001769638	-4.233868494	4.65E-05	0.000154931	0.977116756
G2M_CHECKPOINT	-0.262545334	-0.009652295	-6.607675712	1.27E-09	6.37E-09	11.19256029
E2F_TARGETS	-0.307774711	-0.0117962	-6.984771465	1.97E-10	1.10E-09	13.02673616
UNFOLDED_PROTEIN_RESPONSE	-0.346947533	-0.007152397	-9.79632073	7.71E-17	1.29E-15	27.61714378
MYC_TARGETS_V1	-0.404276848	-0.019148287	-9.594311527	2.29E-16	2.29E-15	26.53774189
MYC_TARGETS_V2	-0.505440577	-0.012602161	-11.06904462	7.96E-20	1.99E-18	34.44206826

GSVA, Gene Set Variation Analysis.

**Table 7:** Results of GSEA for High- and Low-Score Groups in TCGA-PRAD

ID	Set Size	Enrichment Score	NES	*p* value	*p.*adjust	*q* value
KEGG_RIBOSOME	87	0.8122653	3.408139	1e-10	5.52e-09	3.51e-09
WP_CYTOPLASMIC_RIBOSOMAL_PROTEINS	88	0.7881710	3.306846	1e-10	5.52e-09	3.51e-09
REACTOME_SRP_DEPENDENT_COTRANSLATIONAL_PROTEIN_TARGETING_TO_MEMBRANE	113	0.7438685	3.259412	1e-10	5.52e-09	3.51e-09
REACTOME_EUKARYOTIC_TRANSLATION_ELONGATION	93	0.7660769	3.113619	1e-10	5.52e-09	3.51e-09

GSEA, Gene Set Enrichment Analysis; NES, normalized enrichment score. PRAD, Prostate adenocarcinoma.

**Table 8:** Results of GSVA for High- and Low-Score Groups in TCGA-PRAD

Pathway	logFC	AveExpr	*t*	*p* value	adj. *p* value	*B*
APICAL_SURFACE	0.399094387	0.017309712	20.05460213	1.06E-66	5.29E-65	141.0449513
EPITHELIAL_MESENCHYMAL_TRANSITION	0.327245743	-0.00217349	11.96221102	2.65E-29	1.89E-28	55.25956132
APICAL_JUNCTION	0.302307037	0.005083619	15.94537412	6.79E-47	1.70E-45	95.57419816
UV_RESPONSE_DN	0.284131166	-0.000417915	11.85845637	7.05E-29	4.41E-28	54.28812278
MYOGENESIS	0.27786801	0.001792727	11.07286451	9.86E-26	4.48E-25	47.09548188
INFLAMMATORY_RESPONSE	0.256315956	0.003198587	9.66540553	1.95E-20	5.75E-20	35.0017518
KRAS_SIGNALING_UP	0.250665163	0.01160121	11.62346357	6.34E-28	3.52E-27	52.10601491
IL6_JAK_STAT3_SIGNALING	0.246141236	0.000390695	9.151814062	1.27E-18	3.03E-18	30.87017314
COAGULATION	0.23912964	0.005065231	10.63723118	4.81E-24	1.85E-23	43.2381387
COMPLEMENT	0.237221676	0.004742927	11.02577717	1.51E-25	6.28E-25	46.67387432
PEROXISOME	-0.116451325	-0.002291117	-5.387372342	1.09E-07	1.60E-07	6.145516575
FATTY_ACID_METABOLISM	-0.119136263	-0.001140756	-5.033462443	6.65E-07	8.98E-07	4.391777331
MTORC1_SIGNALING	-0.229938131	-0.017130498	-10.00510521	1.14E-21	4.06E-21	37.82045439
G2M_CHECKPOINT	-0.251102746	-0.027619506	-9.257515458	5.45E-19	1.36E-18	31.70735182
OXIDATIVE_PHOSPHORYLATION	-0.252763517	-0.021257836	-8.533876886	1.56E-16	3.25E-16	26.11758926
DNA_REPAIR	-0.26291587	-0.019577602	-11.50712705	1.86E-27	9.32E-27	51.03521086
E2F_TARGETS	-0.282545327	-0.036452145	-9.818968218	5.44E-21	1.81E-20	36.26768167
UNFOLDED_PROTEIN_RESPONSE	-0.287934365	-0.013826155	-13.00800813	1.08E-33	1.08E-32	65.30775346
MYC_TARGETS_V1	-0.398588642	-0.025564717	-14.32183794	1.86E-39	3.09E-38	78.51875244
MYC_TARGETS_V2	-0.419622662	-0.024925861	-13.92405649	1.10E-37	1.37E-36	74.45661342

GSVA, Gene Set Variation Analysis; PRAD, Prostate adenocarcinoma.

## Data Availability

All data generated or analyzed during this study are included in this published article and its supplementary information files. The study utilized pre-existing, publicly available data from TCGA-PRAD, GTEx project, and GEO under accession numbers GSE88808 and GSE21034.
