# Supplementary figures and images for: A Hypoxia and Immune Escape-Related Gene Signature for the Diagnosis of Prostate Cancer: An Integrated Bioinformatics Study
Source: Int J Med Sci. 2026 May 18;23(7):2225–51. doi: 10.7150/ijms.133397 (PMC13280752; doi:10.7150/ijms.133397)

## TCGA-PRAD

significance

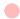

Up

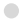

No

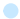

Down

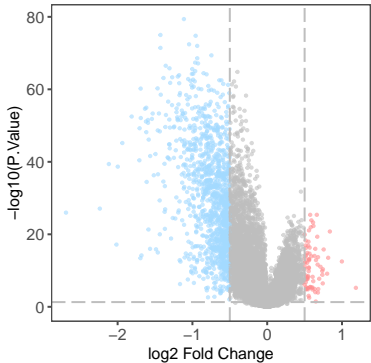

Supplement: Supplementary file 1 — Supplementary figure and tables. [file ijmsv23p2225s1.zip › Supplementary Materials/Figure S1.pdf]
